# Understanding how facilitators adapt to needs of STEM faculty in online learning communities: a case study

**DOI:** 10.1186/s40594-022-00371-x

**Published:** 2022-09-05

**Authors:** Makenna M. Martin, Fred Goldberg, Michael McKean, Edward Price, Chandra Turpen

**Affiliations:** 1 Center for Research in Mathematics and Science Education, San Diego State University, San Diego, CA 92120 USA; 2 Department of Physics, California State University San Marcos, San Marcos, CA 92096 USA; 3 Department of Physics, University of Maryland, College Park, MD 20742 USA

**Keywords:** Faculty Learning Community, Faculty Online Learning Community, Facilitation, Professional growth, Faculty change

## Abstract

**Background:**

Faculty Learning Communities (FLCs) and Faculty Online Learning Communities (FOLCs) are ways to support STEM faculty implementing research-based curricula. In these communities, faculty facilitators take on the role of sharing expertise and promoting discussion. However, as members gain more experience, their needs change from addressing logistical to pedagogical issues. Hence, facilitators need to change their practices in response. However, there is little research on the mechanisms of faculty facilitator change. In this article, we provide a case study of a specific STEM FOLC facilitator and demonstrate the usefulness of a teacher change model to investigate facilitator change.

**Results:**

Guided by our adaptation of the *Interconnected* *Model* *of* *Professional* *Growth* *(IMPG),* we conducted interviews with FOLC facilitators, and selected a case facilitator who reported changes in facilitation goals and strategies over time. The model helped us identify specific areas of change and potential mechanisms for these changes. Using themes of change identified in the case facilitator interview, we developed coding schemes to analyze his FOLC meetings over a 2-year period. We found empirical evidence from multiple data sources, including FOLC meetings and facilitator reflections, that supported the change themes, including: changing his role as an “expert” by sharing his own expertise less and drawing on others’ expertise more frequently, changing his response to members’ comments by jumping in to answer less frequently and withholding his own responses more often to encourage member sharing, and a change in group discussions towards less logistical and more pedagogical conversations.

**Conclusions:**

Our findings suggest that the IMPG can be fruitfully adapted to study facilitator change. A diagrammatic representation of the IMPG provides a description the types of change the case facilitator experienced and the factors that supported those changes. We discuss how the methodology used to analyze facilitator actions in FOLC group meetings may be useful to study other types of professional growth. Finally, because our analytical model allowed us to identify mechanisms of facilitator change, we describe the implications and provide suggestions to support facilitators in other faculty community groups.

**Supplementary Information:**

The online version contains supplementary material available at 10.1186/s40594-022-00371-x.

## Introduction

### Faculty learning communities

In the past two decades, faculty learning communities (FLCs) have emerged as an important mechanism for higher education faculty development in effective implementation of pedagogical innovation (Cox, 2004). FLCs are groups of approximately ten faculty in related disciplines (for example, physics and physical science) that meet regularly to engage with issues in teaching and learning. Facilitators of FLCs may be faculty development professionals or interested faculty members. More recently, faculty online learning communities (FOLCs) have been used as geographically distributed, discipline-specific FLCs that support and provide resources for faculty implementing Research-Based Instructional Strategies (RBIS) to enhance student learning (Cox, 2004; Dancy et al., 2019; Price et al., 2021). Facilitators use various strategies to achieve the goals of their communities alongside the needs of the faculty members (Andrews-Larson et al., 2017; Lau et al., 2018; Ortquist-Ahrens & Torosyan, 2009; van der Want & Meirink, 2020; Zhang et al., 2011). This article will broadly explore the ways that facilitator goals and strategies can change over time and possible change mechanisms. The findings will help fill a gap in the literature, as research has yet to explore how faculty facilitators’ views and practice of their role changes over time. The findings also provide insights into how faculty facilitator growth can be supported.

Student-focused, active learning, or other RBIS have demonstrated efficacy in improving student learning outcomes, including in undergraduate STEM (Freeman, 2014; Paolini, 2015). However, university faculty who utilize these strategies report a variety of challenges, requiring support to foster continued use of them (Henderson et al., 2007; Henderson et al., 2012). One of the recommendations to assist faculty in implementing RBIS is to provide on-going, people-based support (Henderson et al., 2015), such as FLCs and FOLCs (Corrales et al., 2020; Price et al., 2021). In these communities, faculty can share strategies, materials, and help adapt pedagogical strategies and curricula to each other’s unique teaching contexts and needs (Elliot et al., 2016). Evidence indicates that these groups help faculty to persist in using RBIS (Corrales et al., 2020; Price et al., 2021; Rundquist et al., 2015). However, to attain these benefits, community meetings require structure to encourage and promote productive faculty conversations. To do so, FLCs and FOLCs are typically designed with one or two faculty facilitators (Cox, 2004; Dancy et al., 2019), whose actions can be crucial to faculty’s opportunities to learn from these kinds of meetings (Andrews-Larson et al., 2017).

### Facilitation

In many kinds of professional development, facilitators are important to teacher learning; even in highly structured meetings, the absence of an experienced facilitator can lead to weaker learning outcomes (Allen & Blythe, 2018). Facilitators must take on multiple roles, drawing on their subject-specific classroom teaching expertise while also utilizing strategies to promote the professional learning of others (Perry & Boylan, 2018). The facilitator sets the tone of the conversation and helps structure how the participants interact; thus, the methods of experienced facilitators can lead to greater professional growth within peer groups (Allen & Blythe, 2018; Ortquist-Ahrens & Torosyan, 2009). In typical FLCs and FOLCs, facilitators are tasked with the role of encouraging faculty participants to share their own ideas and experiences, as well as helping address the concerns of members, whether by sharing their own experiences or drawing on other participants (Cox, 2004; Dancy et al., 2019). The facilitator helps enable conversation that is productive for the goals of the meeting and for the needs of the participants (Ortquist-Ahrens & Torosyan, 2009). While there has been research on facilitation in K-12 teacher workgroup meetings (e.g., Allen & Blythe, 2018; Andrews-Larson et al., 2017; Schwarts, 2020; Zhang et al., 2011) and university faculty facilitating student activities (Brody & Hadar, 2016; Brown et al., 2018; Hmelo-Silver & Barrows, 2006, 2008), there exists little descriptive research on facilitators in higher education FLCs and FOLCs (Ortquist-Ahrens & Torosyan, 2009), despite the importance of facilitators these settings.

This article focuses on facilitator change, which may occur due to the evolving needs of the community or development in the facilitator. Faculty implementing an RBIS will have different needs as they gain more experience. The Concerns-Based Adoption Model (CBAM) describes the needs of educators at various stages of adopting a new teaching strategy, suggesting that educators’ needs change over time (Anderson, 1997). Educators may initially focus on basic concerns, such as learning what the strategy is and how it works, and then gradually shift their attention to more complex concerns, such as if the strategy is working effectively or could be modified or expanded. Thus, in learning communities centered around RBIS, faculty will likely initially have more concerns that are logistical and practical, but are likely to change over time and become more pedagogical. Indeed, this kind of change towards more reflective thinking around issues of teaching and learning has been observed in studies of teacher change (e.g., Jiang et al., 2021) and more specifically within FOLCs (e.g., Corrales et al., 2020; Dancy et al., 2019). This shift suggests that facilitators may need to adjust their practice according to changing needs. Petrone and Ortquist-Ahrens (2004) suggest that it is *necessary* for facilitation practice to change, suggesting that FLC facilitators should seek to minimize their own leadership role over time to allow group members to take on more agency.

University faculty facilitating FLCs or FOLCs may also change over time as they gain experience with facilitation. Typically, university faculty members are recruited as facilitators without significant prior experience or training as peer facilitators (e.g., Dancy et al., 2019). An earlier study by Sandell et al. (2004) found that 40% of university facilitators are not given training for the role and the 60% provided with preparation are typically only given readings or one-time workshops. Although faculty may be content and/or curriculum experts, they are not formally prepared to be facilitators of peer learning communities. This is not to say that faculty have no expertise to draw from to fulfill their role; they may have previous experiences facilitating classroom discussions, faculty meetings, committee responsibilities, or other professional activities (Ortquist-Ahrens & Torosyan, 2009; Sandell et al., 2004). Also, in some long-term FLCs or FOLCs, faculty who start out as members of the community may be asked to take on a facilitator role in ensuing semesters. In that case, they can draw on observations of previous facilitator(s) as a resource for their own facilitation (Dancy et. al. 2019). However, facilitators in FLCs and FOLCs are charged with aiding others in developing professional knowledge, which may require skills outside of those learned or observed in other settings.

Of the handful of studies on STEM FLCs and FOLCs at the university level (e.g., Corrales et al., 2020; Dancy et al., 2019; Elliot et al., 2016; Price et al., 2021; Tinnell et al., 2019), only one brief paper focuses on facilitation (Lau et al., 2018). We are not aware of any literature examining how faculty members approach their facilitation role, nor how and why they change their practice over time as the needs of the learning community change. Yet, because of the importance of FLCs and FOLCs to support faculty learning, both facilitators and organizers of learning communities could potentially learn from a detailed study to guide their planning.

This article presents a case study of a faculty member who takes on the role of facilitator in a multi-year FOLC. Our research goals are to describe how a facilitator’s goals and strategies changed over two years in this role, and to point to the specific factors that seemed to contribute to those changes. We aim to inform both the research and professional development communities about how FLC/FOLC facilitators might be expected to change and how these changes might be supported. In the next section, we focus on the conceptual framework that guides our description and explanation of facilitator change, an adaptation of the Interconnected Model of Professional Growth (IMPG; Clarke & Hollingsworth, 2002). We also describe the context of our study and our case study facilitator. The methods section details the emergent change themes about how his goals and strategies shifted over time, and the data sources and analytical methods we used to support those themes. We then describe the results of our analyses and how the results can be represented using the IMPG. Finally, we discuss how our study provides insights into the preparation and evolution of university faculty as FLC or FOLC facilitators.

### Conceptual framework

Our focus in this study is on facilitator change. Even with related prior experiences, such as teaching, most facilitators have limited specific preparation for the role. Thus, we expect that many facilitators grow and change as they gain experience. By understanding facilitators’ growth processes, we seek lessons for improving their preparation and support. To describe and better understand the mechanisms of change in professional practice, Clarke and Hollingsworth (2002) developed the Interconnected Model of Professional Growth (IMPG). The model has typically been applied to teacher change, but has been successfully adapted to describe and understand facilitator change (e.g., Perry & Boylan, 2014, 2018). Like Perry and Boylan (2018), we adapt the Interconnected Model of Professional Growth (IMPG) to study facilitator change.

The IMPG builds on prior linear models (e.g., Clarke & Peter, 1993; Guskey, 1986) to account for more complex mechanisms of growth by incorporating multiple domains of influence and different ways change can occur (Clarke & Hollingsworth, 2002). The model conceptualizes teachers’ professional growth as a type of learning, drawing on empirical data of teacher change and on learning theories, including the Community of Practice framework (Wenger, 1998). This framework considers individuals’ learning within a social group, such as a community of teachers; practitioners evolve through their own practice as well as by their interactions with others. Thus, to account for these multiple influences on learning, the IMPG looks at change in terms of four domains of teacher experience—external, practice, consequence, and personal—which collectively comprise the *change* *environment*.

In our adapted IMPG, the *domain* *of* *practice* encompasses actions facilitators take to perform their role, including changes in strategies they implement during meetings (Perry & Boylan, 2018). The *domain* *of* *consequence* includes salient outcomes, such as their observations and inferences about what happened during meetings. The *personal* *domain* captures their knowledge and beliefs about facilitation, including changes in their goals for their facilitation efforts (Perry & Boylan, 2018). Like Perry and Boylan (2018), we reconceptualized the *external* *domain* as the “social” domain to account for the influence of facilitators’ colleagues and peers. We further include influences from co-facilitators or project staff, and teaching experiences, which all originate outside FOLC group meetings. It is important to note that these domains represent distinct types of *changes*, and that change within one domain can influence change in a separate domain.

To describe the mechanisms of professional learning, the four domains can be connected by the mediating processes of *enactment* and *reflection*. *Enactment* is when a teacher or facilitator implements a *new* practice informed by changes in one of the other three domains; this is different from acting on an *existing* belief or idea, as the latter is represented by a change in the domain of practice (Clarke & Hollingsworth, 2002). The mediating process of *reflection* is when thinking about changes in one domain leads to change in another domain. For example, an IMPG diagram with a dashed *reflection* arrow pointing from the domain of consequence to the personal domain means that reflecting on the change in outcome lead to a change in knowledge, beliefs, or attitude. A solid *enactment* arrow pointing from the personal domain to the domain of practice means that actual changes in knowledge, beliefs, or attitudes caused (enacted) a change in practice.

**Fig. 1:**
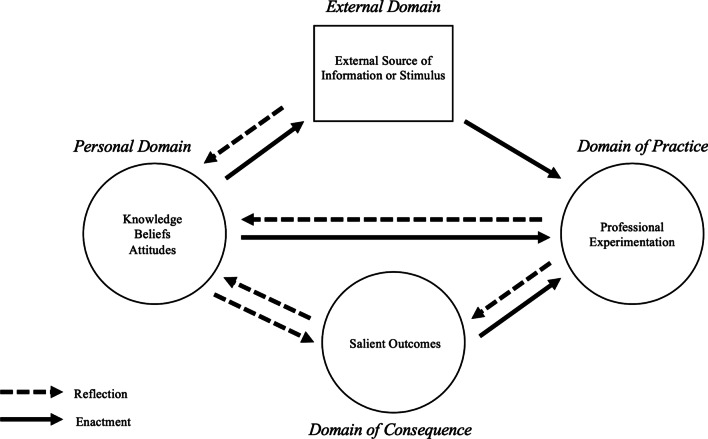
The adapted Interconnected Model of Professional Growth. IMPG figure adapted from Clarke and Hollingsworth (2002), Perry and Boylan (2018)

The base diagram (Fig. 1) represents the possibilities for change pathways; IMPG diagrams are created for individuals and may or may not include all domains or connection arrows; the diagram may be simple, with few domains and arrows, or more complex based on the intricacy of the mechanism of change. The connections between domains may occur in a particular order and can be represented with numbered arrows that illustrate an individual’s *change* *sequence*. A change sequence is a connection between at least two domains, where information about the domains and the mediating connection arrows is based on empirical data. A change sequence can lead to professional *growth* if it produces long-lasting effects.

### The current study

Using the adapted IMPG, we account for facilitator change by considering changes within the four domains and the reflection and enactment connections between them. Using the model as our guide, we aim to address the following two research questions:

**RQ1**: How do a FOLC facilitator’s goals and strategies change over multiple semesters in the FOLC?

**RQ2**: What factors seem to influence the changes in the facilitator goals and strategies?

We intend to answer these questions by constructing an IMPG diagram for our case study facilitator. Changes described in the *personal* *domain* and *domain* *of* *practice* of the IMPG will directly address our first research question involving changes in facilitator’s goals and strategies. After creating an IMPG model of a facilitator’s change over time, entries in the *external/social* *domain* and changes in the *domain* *of* *consequence*, plus our interpretation of the *enactment* and *reflection* arrows connecting all the domains, will together address our second research question on the mechanisms of change. Answering these research questions can provide insights into how a particular FOLC facilitator adapted to changing needs of their community, as well as helping to understand the process of facilitation and the experiences that enabled facilitator change.

### Study context

#### Next Gen PET faculty online learning community

**Fig. 2:**
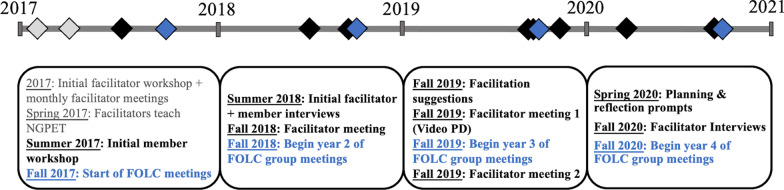
Timeline of NGPET FOLC group member and facilitator activities. *Note.* The events in blue represent the successive years of the FOLC group meetings. The events in black represent facilitator and FOLC member activities (those in grey represent activities where the case facilitator was not present)

The context of this study is the Next Gen PET faculty online learning community (NGPET FOLC; Price et al., 2021) consists of approximately 50 faculty who use the Next-Generation Physical Science and Everyday Thinking curriculum (NGPET) (Goldberg, 2015) to teach physics or physical science to future elementary teachers or general education students. The NGPET curriculum is a student-focused, hands-on, guided inquiry curriculum, and its implementation can challenge faculty who are only experienced teaching science in a traditional lecture format (Goldberg et. al., 2010; Price et al., 2021). The NGPET FOLC was established in 2017, to help support faculty implementing the NGPET curriculum and to promote reflective practice and professional growth (Price et al., 2021), and has continued through 2021 (and beyond). Figure 2 lists the major activities during the first 4 years of the NGPET FOLC.

The community is divided into separate FOLC groups of 8–12 faculty, each led by 2–3 faculty facilitators who guided discussions and provided curriculum expertise. A fifth FOLC group was added in the fall of 2018, consisting of faculty new to the community, with one existing community member “promoted” to a facilitator role to help guide the new FOLC group alongside one original facilitator. The FOLC groups met bi-weekly online via Zoom video conferencing each semester to discuss issues related to pedagogy and the NGPET curriculum implementation. The membership of the groups changed each semester based on faculty availability. All meetings were recorded for potential analysis by project staff. During the summer of 2018, project staff interviewed the facilitators and selected FOLC group members to probe their experiences during the first year of the project.

#### NGPET FOLC facilitators

The FOLC group facilitators were initially chosen for their facilitation role because they had extensive experience teaching either NGPET or one of its predecessors. Prior to their involvement in the NGPET FOLC, the facilitators had no prior experience facilitating FOLCs, although they did have experience facilitating student discussions in the NGPET classroom, and some had experience facilitating faculty meetings at their institutions. It was reasonable to expect that some of those skills could serve as productive resources for facilitating faculty discussions in the FOLC (Borko et al., 2014; Ortquist-Ahrens & Torosyan, 2009; Tekkhumru-Kisa & Stein, 2017). As facilitators of FOLC meetings, they were tasked with multiple roles: helping members to overcome challenges by relating their own experiences and helping to manage and promote group discussion to encourage sensemaking around problems of practice (Ortquist-Ahrens & Torosyan, 2009). The facilitators were provided with a brief introduction to facilitation at the very beginning of the project (described below), but primarily honed their facilitation skills over time as they both led FOLC group meetings and met periodically with the other facilitators and project staff to discuss facilitation issues.

The project team conducted some explicit professional development activities to help the facilitators become more successful. Figure 2 lists these professional development activities. In spring of 2017, the facilitators met for a 2-day workshop to introduce them to the NGPET curriculum and to review some of the research on supporting instructional change (Henderson et al., 2010, 2012). They reviewed some known barriers to the use of RBIS and were provided with strategies for developing a community that engages in productive discussions about pedagogy. The facilitators then met monthly with project staff via Zoom video conference to discuss NGPET implementation obstacles, discuss facilitation responsibilities, and share facilitation challenges and other issues that arose in their FOLC group meetings. During the 2018–2019 academic year, the facilitators met just once with project staff to discuss facilitation issues.

In fall 2019, the project team made a concerted effort to provide PD for the facilitators. The project team sent a brief paper on facilitation suggestions, based in part on work being done by others in our research group, and transcripts of two conversations involving a facilitator and group members from a similar FOLC (Dancy et al., 2019; Lau et al., 2021; see Additional file 1). At the fall 2019 meeting, the suggestions were discussed, including guidance by project staff with a particular focus on the idea of *turning* *towards* a problem of practice (Horn & Little, 2010). During one meeting, based on previous research on video-based professional development (e.g., Borko et al., 2011, 2014; Tekkumru-Kisa & Stein, 2017), the facilitators were shown video clips of the transcripts they had read, were asked what they noticed the facilitator doing, and if they would have done anything differently. This led to substantive discussions with facilitators reflecting on their own facilitation experiences the previous year and thinking about strategies they would try to implement going forward. At the end of fall 2019, the facilitators met for a second time, when they were prompted to share specific examples where facilitation went well, or they felt like they were able to put the suggestions into practice. This generated another substantial conversation where several examples were discussed. The PD and facilitator discussions lead to the development of facilitator interviews and reflections prompts that are discussed in the following section.

## Methodology

We utilized a case study approach to investigate facilitator change in the context of the NGPET FOLC (Creswell & Poth, 2017). We first discuss the design of facilitator interviews utilizing the IMPG framework. Next, we introduce the facilitator who is the focus of this case study, describing his background and experience within the NGPET FOLC. We then describe themes that we identified in his interview, representing how his facilitation changed over time. Finally, we describe the methods we used to analyze the meetings he facilitated around the identified themes.

### Facilitator interviews and reflections

Facilitators and members were initially interviewed in the summer of 2018 with the goal of learning more about the group meetings and NGPET implementation issues (Price et al., 2021). The project staff also interviewed facilitators to provide them an opportunity to reflect on their first year of facilitation. After the PD implemented in fall 2019, the project staff also developed a planning/reflection form (see Additional file 2) that was distributed to facilitators in spring 2020. The form consisted of a series of prompts for the FOLC group co-facilitators to use in planning and reflecting on each meeting, listing their goals for the meeting, and any strategies they thought useful to try to accomplish those goals (Hands et al., 2015; Hmelo-Silver & Barrows, 2006; Patton et al., 2013; Zhang et al., 2011). In the reflection section, facilitators were asked how they thought the meeting went, and to what extent their initial goals were accomplished. Project staff had access to the responses to monitor facilitator change. Information from the forms, the facilitation guidelines distributed the previous semester, and the IMPG were used to design facilitator interviews administered in fall 2020.

In fall 2020, second facilitator interviews were conducted using a protocol designed using the IMPG change framework, providing another reflection opportunity for facilitators. The second interviews focused on how their goals and strategies *changed* over the duration of the project and were designed to elicit information that could be used to empirically inform the description of IMPG domains and their connections (see Fig. 1). Table 1 shows the interview questions and the domains that the questions addressed (full protocol available in Additional file 3). The interview questions helped gather data about the domains of the individuals, and the sub-questions about change helped develop the causal mechanisms for change. From this information, we could develop a possible change narrative for the facilitator.

**Table 1:** Interview questions and the IMPG domain addressed

Interview question	Domain addressed			
	Personal domain	Domain of practice	Domain of conseq	External domain
1. What do you see as your role as a facilitator? a. How has this changed over the course of the project?	X	X		
2. What are your goals as a facilitator? a. How has this changed over the course of the project?	X	X		
3. What actions or strategies did you use to achieve your goals? a. How has this changed over the course of the project?		X	X	
4. How successful do you think you were in achieving your facilitation goals?		X	X	
5. How did your co-facilitator and/or group members help you to achieve your goals?	X		X	X
6. Over the course of your involvement, what has helped you become a more effective facilitator?		X	X	X
7. What advice would you give to new facilitators? a. How could we better support facilitators?		X	X	X

A copy of the full interview protocol is available in Additional file 3

### Case study facilitator: Craig

Craig was initially chosen to join the NGPET FOLC as a regular participant. He stated on his FOLC application that he had taught an NGPET predecessor curricula for several years, and thus was very comfortable and familiar with implementing the student-focused pedagogy. He had a Ph.D. in physics, taught physics courses at a university, and had experience in physics education research. Craig was interviewed in summer 2018 (see Fig. 2) about his experience teaching Next Gen PET and his perceptions of the focus of the group meetings and the facilitators’ role. At the end of the first year, an additional FOLC group was formed to accommodate new faculty who wanted to join the FOLC. Craig was asked to facilitate due to his substantive and thoughtful contributions to his group’s discussions in his first year as a member. He was teamed with an experienced co-facilitator from the first year. For the following two years Craig co-facilitated three different FOLC groups and participated in all the PD activities organized by the project staff (Fig. 2).

During his fall 2020 facilitator interview, Craig described substantive changes in his facilitation goals and strategies during his experience as a facilitator, and clearly articulated reasons for those changes. His comments provided us with sufficient information to develop a comprehensive story of change in terms of the four IMPG domains and the reflection and enactment connections between them. While interviews with other facilitators also revealed potential stories of change, they were not as substantive as Craig’s, consequently, we decided to focus on him as our single case facilitator.

**Fig. 3:**
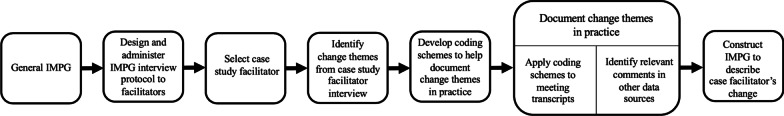
An overview of the methodological process of the study

Our strategy for developing the case study was to first identify change themes in his interview and then develop coding schemes to analyze meetings he facilitated, to document how those themes played out in practice. We also used thematic analysis of multiple data sources to identify descriptive statements related to the IMPG that showed how Craig’s intentions or ideas about facilitation emerged and changed (Braun & Clarke, 2006). Figure 3 provides an overview of our methodological approach and Table 2 describes our data sources and analyses.

**Table 2:** Data sources and analyses applied during the study

Data source (transcript)	# Analyzed	Analysis performed
2018 member interview	1	Thematic analysis of IMPG-related change claims
2020 facilitator interview	1	Established IMPG-related change claims
FOLC group meetings 2018–2020	10	Facilitator vs. Participant Focused response coding scheme Segment Sequence coding scheme Segment Content coding scheme Thematic analysis of IMPG-related change claims
Facilitator–project staff meetings	3	Thematic analysis of IMPG-related change claims
Planning and reflection with co-facilitator	2	Thematic analysis of IMPG-related change claims

### Change themes

Table 3 describes three change themes identified from Craig’s facilitation interview, along with an example supporting comment for each theme. Craig’s interview transcript and additional supporting comments are available in Additional file 5, Additional file 6: Table S1. The themes focus on how his facilitation role changed, how the way he responded to issues raised by participants changed, and how the content of his meetings changed over time. It is important to note that, although these themes were tailored to Craig, at least the first and third themes are like changes that would be expected based on the Concerns-Based Adoption Model (Anderson, 1997).

**Table 3:** Three change themes with example supporting quote from case facilitator interview

Change theme	Example supporting quote from 2020 interview
Role change He originally believed his role was to draw on his expertise by sharing his own experience with the group. Later, he believed that his role should be to share his experience less frequently and draw on members’ ideas and experiences more often	“What I've learned is I've got to keep myself in check and make sure that I'm not dominating the conversation. Sometimes that means that even if I've got a great idea for the mystery tube, I actually don't get to share it”
Response Change He changed his practice by jumping-in to address issues raised less often, and withholding his own response more frequently, to encourage member sharing	“We kind of have to put ourselves, our teacher mode in check, because we so often, we've got things that we want to share and, ‘Oh yeah, I've seen this before and this is what I did, and this is how I solve that problem’… So, we've really gotta hold back on that as a facilitator […], we need to give everyone space to talk […] I'm more trying to get other people to talk about what it is that they've done”
Meeting content change He observed that initially the FOLC group discussions were more logistical in nature, whereas later the group members could engage in more pedagogical discussion. Consequently, he changed his practice to promote opportunities for pedagogical conversation	“Initially I think it was very nuts and bolts and logistical. […] There was discussion about pedagogy as well, but I think more so at the beginning than now we really paid attention to logistical things and details to help iron out. Now everyone who's in the project for the most part is pretty experienced with the curriculum. So that's less of an issue”

Full interview transcript and additional supporting quotes for the change themes can be found in Additional file 5, Additional file 6: Table S1

### FOLC group meeting analysis

To put our description of Craig’s changes in context, we identified some of his meetings as ‘early’ in his facilitation practice and some as ‘later’. Because of the PD intervention at the beginning of Craig’s second year as facilitator (see Fig. 2), we define his *early* *meetings* as occurring during his first year of facilitating (fall 2018–spring 2019) and his *later* *meetings* as occurring during his second year (fall 2019–spring 2020). Because the COVID-19 Pandemic shifted all instruction online, thereby significantly changing the focus of group meetings, we ended our analysis in the middle of spring 2020.

The composition and attendance of the meetings varied by semester. We used the following criteria to select meetings to analyze in detail: Craig was either sole facilitator or co-facilitator at the meeting and there were at least three faculty members present. Applying these criteria resulted in the inclusion of five early meetings and five later meetings. These meetings were transcribed for analysis, using coding schemes to determine the extent to which Craig’s change themes were demonstrated in practice (see Table 2).

For our coding approaches, we used a combination of a priori and inductive coding. For each of the coding schemes, we started with a subset of codes based on prior analyses reported in the literature and monitoring of FOLC meetings by the research team. Codes were then modified as necessary as the data were reviewed. We next describe the coding schemes developed to analyze meetings around the three themes (see Table 3).

### Role change

To address this theme, we first segmented meetings by shifts in conversational topic into those focusing on issues of teaching and learning, and those not, and only further analyzed the former. Each segment begins with a prompt posed as a question or topic (See Additional file 4 for transcript segmentation and preliminary analysis). Responses were identified, distinguishing between facilitator and other participant contributions. We then developed the *Participant* *focused* vs. *Facilitator* *focused* response coding scheme. Following others (e.g., van der Want & Meirink, 2020), this scheme categorized Craig’s responses during the meeting as either *participant* *focused*, *facilitator* *focused*, or other. *Participant* *focused* responses are those that elicit information or experience from group members or developing on others’ ideas, while *facilitator* *focused* responses are when the facilitator shares their own experiences or expertise (see Table 4). Subcodes are used to describe specific types of responses based on a combination of existing codes from the literature (e.g., Andrews-Larson et al., 2017; Zhang et al., 2011) and inductive coding of the meeting data, including sharing experience, providing solutions or information, making a pedagogical statement, asking a question, revoicing, and summarizing.

**Table 4:** Facilitator focused vs. participant focused coding scheme

Code	Description
Facilitator Focused	Responses focused on sharing the expertise of the facilitator
Experience	Facilitator shares personal experience, describes classroom situations. E.g., describing how students responded to a particular activity
Solutions	Facilitator offers a solution or multiple solutions for a problem of practice. E.g., suggesting a method for forming groups
Information	Facilitator provides information, resources, or a status update. E.g., giving details about where to find videos, information about syllabi, etc.
Pedagogical statement	Facilitator makes a pedagogical observation or describes a formal concept. E.g., “Students struggle with wanting to know the right answer, so you should set expectations at the beginning of the course”
Participant Focused	Responses focused on building upon or drawing out others’ ideas
Logistics, status update, or clarification question	Facilitator asks a question related to logistics, asks a member to report about the status of their class, or asks a clarifying question following up on a member’s comment. E.g., “How many units do you teach per semester?”
Redirecting question	Facilitator asks others to respond to a question or issue raised by others. E.g., “Tom uses that activity. Tom, can you tell us how you use it in class?”
Revoicing	Facilitator revoices comments or ideas shared by group members. E.g., “So, I hear you say that xxx.”
Summarizing	Facilitator summarizes ideas or comments shared by group members with pedagogical intent. E.g., “So, we have two suggestions to consider, one is xxx and the other yyy.”
Pedagogical question	Facilitator asks a pedagogical question to the group. E.g., “How do you promote critical dialogue in classes with low attendance?”
Other	Any response that doesn’t fit into the previous categories. E.g., compliments, making a meta-comment about the FOLC, making a general comment, summarizing for late joiners (not for a pedagogical reason)

### Response change

For this theme, we developed the *segment* *sequence* coding scheme, which built on Lau et al. (2018) idea of *withholding,* when a facilitator does not immediately offer their opinion or try to solve a problem (Lau et al., 2018). For each meeting segment we identified *when* Craig responded to a probe (question raised by participant); did he “jump in” to answer with his own experience, or did he “withhold” his own response and/or encourage others to respond first? (Table 5).

**Table 5:** Segment sequence coding scheme

Code	Description
Jump In	Group member offers a probe, and the facilitator provides a Facilitator-Focused response before participants can provide a substantive response
Withholding	Someone else offers a probe and then another person provides a substantive response before the facilitator provides a response
Other	Any situation that does not fit into the previous categories. E.g., someone asks a direct question to the facilitator and the facilitator immediately responds; facilitator responds right away, but with an “other” response

### Meeting content change

Based on prior work of categorizing types of teaching and learning conversations (Horn et al., 2017; Lau et al., 2021), we developed the *segment* *content* coding scheme to categorize segments as pedagogical, logistical or practical, a status update, or other (Table 6).

**Table 6:** Segment content coding scheme

Code	Description
Status update	A report of teaching practice. E.g., “How are things going?”, “Where are you at in the curriculum?”, or “Anything you want to discuss?”
Logistical/practical	Talk related to how members do things in their NGPET course/classroom; why they make certain choices about the NGPET curriculum; general information. No explicit focus on issues of student learning; does not include conversations that lead to substantial pedagogical rationale for decisions
Pedagogical	Conversations specifically talking about issues of student learning or the impact/effect on teaching and/or learning processes. E.g., providing explanation of student thinking in relation to difficulties learning a physics concept, or providing a pedagogical rationale for why certain things are done a particular way in their classroom
Other	Does not fit into other categories. E.g., explanation of a physics concept related to the curriculum without connecting it to student learning or teaching

To ensure that the coding schemes for analyzing the three themes were accurately and consistently applied, three members of the research team collaborated to perform multiple rounds of coding on all ten meetings analyzed. For all the coding schemes, one member did the initial coding, which was checked by a second member, and then blind-coded by a third member. The blind codes were compared to the initial codes, and differences were discussed and reconciled among all three researchers. The coding schemes all had a high degree of fidelity across multiple coders. We then determined the frequency of codes applied to look for patterns across early and later meetings.

In the spirit of checking the credibility of our results and interpretations described in the next sections, we asked the case facilitator to read a near-final draft of this article and to verify if our narrative was consistent with his recollection. He responded and made many comments regarding the compatibility of our observations with his own experience, for example noting that, “Often while reading, I would jot something down or have a thought, only to see it in print immediately afterward.... This indicates to me strong coherence between the manuscript and my experiences.” Specifically, he confirmed that the three change themes we gleaned from his interview, and observed in the meeting data, matched his own perceptions of his changes and the changes within the community.

## Results and discussion

To answer our research questions, we analyzed Craig’s interviews, FOLC group meetings, planning and reflection discussions, and meetings with other facilitators and project staff. Craig’s comments during interviews and facilitator meetings provide information about changes in his beliefs and knowledge represented in the *personal* *domain*. Information provided by the project staff and comments he made about external influences provide information about the *external/social* *domain*. The results of our analyses, along with quotes from FOLC group meetings, facilitator meetings and the fall 2020 interview give us information about Craig’s facilitation practices within meetings, informing the *domain* *of* *practice,* and how he perceived the group meetings went, informing the *domain* *of* *consequence*. We use all the information and analyses to create an IMPG diagram of Craig’s facilitation changes as the last step in our methodological process (Fig. 3). Below we describe the results of our analyses, discuss their implications for describing and understanding Craig’s changes in practice, represent those changes in an IMPG diagram, and finally interpret the diagram to provide answers to our two research questions. The analyses consist of observing trends in the results from applying the coding schemes; however, the results are not intended as formal, statistical claims to be generalized to a broader population.

### Role change

**Fig. 4:**
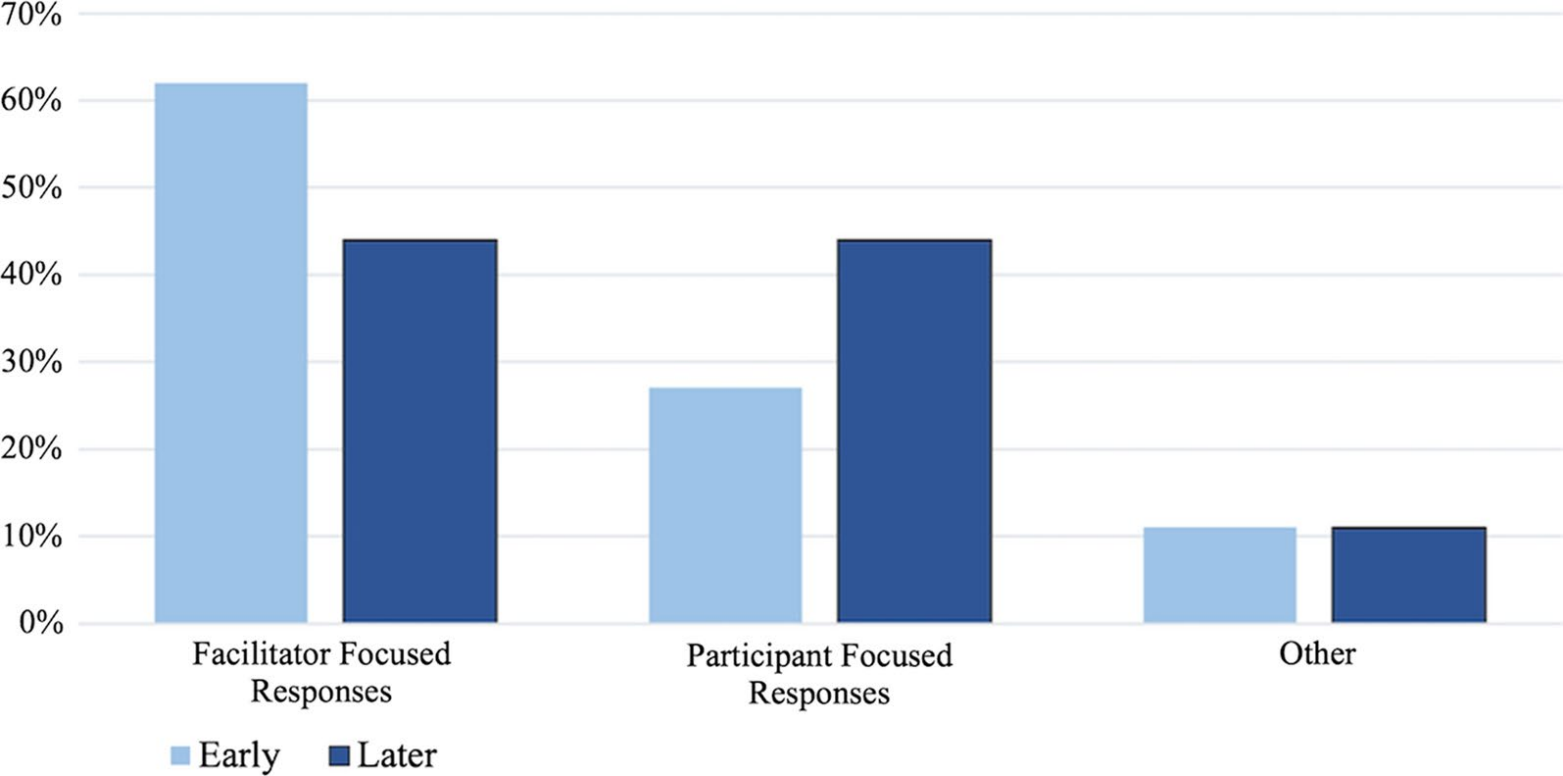
Total Participant Focused, Facilitator Focused, and Other responses in early and later meetings. For the 5 early meetings, there was an average of 23 responses per meeting (averaging roughly 15 facilitator focused, 7 participant focused, and 1 other) and for the five later meetings there was an average of 20 responses per meeting (averaging roughly 9 facilitator focused, 9 participant focused, and 2 other)

Between early and later meetings, there was a decrease in the average proportion of *facilitator* *focused* responses and an increase in the average proportion of *participant* *focused* responses per meeting between early and later meetings (Fig. 4). These changes are consistent with Craig’s role change theme.

**Fig. 5:**
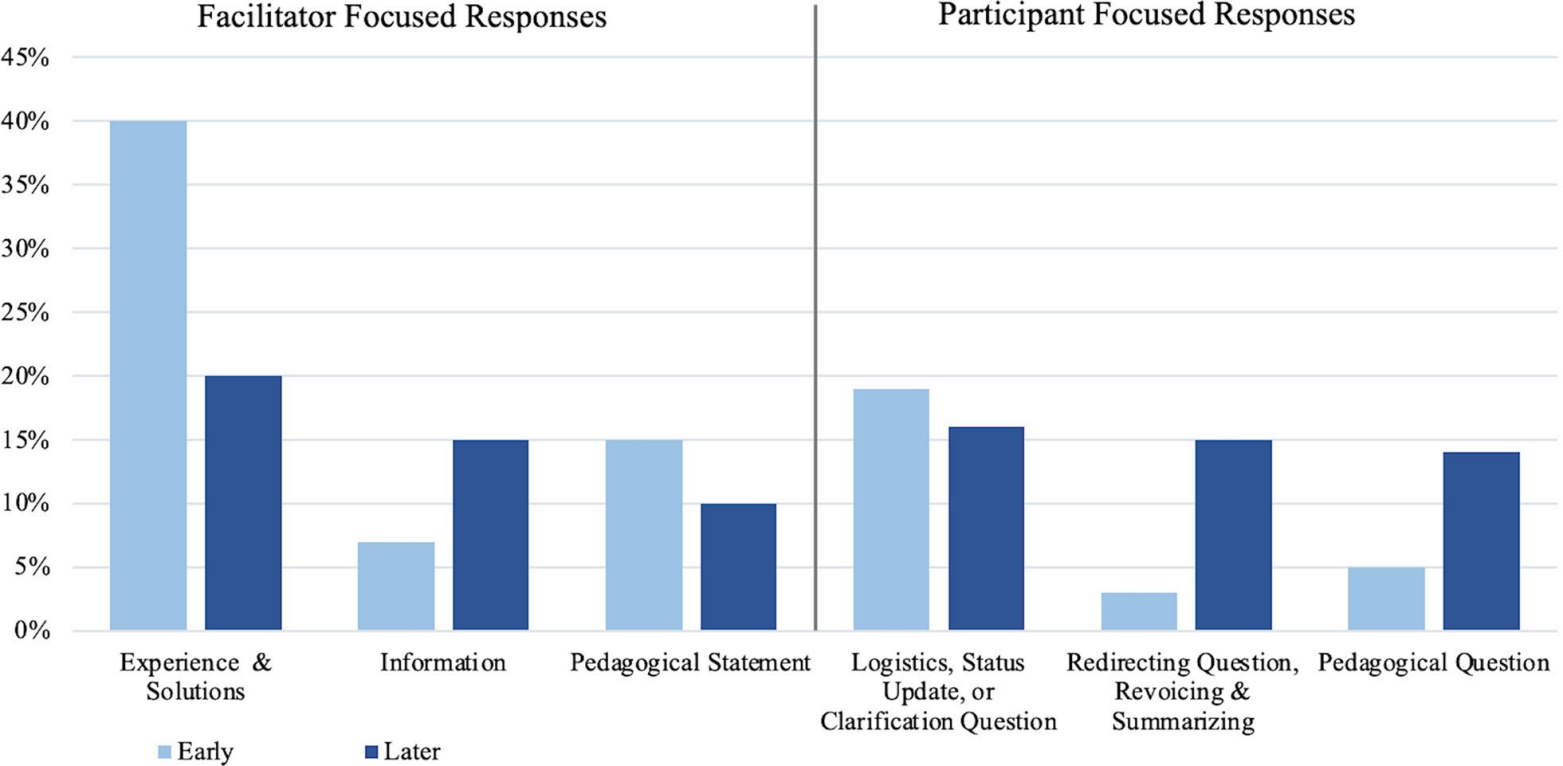
Proportion of Participant Focused and Facilitator Focused subcodes in early and later meetings. For the early meetings, there was an average of 23 responses per meeting, averaging roughly 15 facilitator focused responses and 7 participant focused responses. For the later meetings there was an average of 20 responses per meeting, averaging roughly 9 facilitator focused and 9 participant focused responses. This graph does not include responses coded as *other*

Looking at the subcodes gives us more support for this change theme. Craig responded less frequently with his own ideas and more frequently responded in ways that prompted member sharing. There was a decrease in the average proportion of *experience* and *solutions* subcodes and an increase in *redirecting* *questions*, *revoicing* *comments,* *summarizing* *responses,* and *pedagogical* *questions* per meeting between early and later time periods (Fig. 5).

This change in his role towards more *participant* *focused* responses shown in Figs. 4 and 5 means that he made more attempts to draw out and build upon members' ideas. For example, in later FOLC group meetings Craig shows great finesse in responding to group members’ comments to keep the conversation flowing and encourage participation, as exemplified in a series of responses from a later group meeting (10/18/2019 FOLC meeting):

[00:17:36] “So there's a lot in what you said. Because it sounds an awful lot like standards-based grading or assessment [*Revoice*]. But there might be elements there that are similar to that, I think we can have a conversation about that. But I'm really most interested in thinking about, or talking about, or seeing what other people have to say about the rationale. What's motivating you and your colleagues to try this in intro physics? And does that transfer to the NGPET audience? [*Pedagogical* *Question*] . . . Do you think it's as critical, more critical or not as critical?” [1 Member responds]

[00:20:52] “So has anybody else tried something similar to this in NGPET? [*Redirecting* *question*]” [2 Members respond]

[00:21:55] “Let's hear what are other people's thoughts on this? [*Redirecting* *question*]” [1 Member responds]

[00:24:15] “So [members who haven’t shared], any other suggestions about ideas as to how something like this might be a fit for PET or . . . ? [*Redirecting* *question*]” [2 Members respond and discuss]

[00:35:26] “Any other thoughts or discussion?”

In this excerpt from a later FOLC group meeting discussion, Craig makes a series of comments that demonstrate his efforts to *revoice* ideas shared within the group and repeatedly *redirect* questions to different group members to encourage sharing and discussion. Both the coding and Craig’s statements support the idea that Craig was sharing his own experiences and solutions less, while making responses that encouraged others to share. Craig spoke about this change in his intent or goal directly in his 2020 interview (Table 3; see Additional file 6: Table S1 for additional supporting quotes). Craig mentions that he has been trying to shift his role by sharing his own experiences less and drawing on group members more; now that the members have implemented the curriculum, he believes they can talk more about what they have done.

**Fig. 6:**
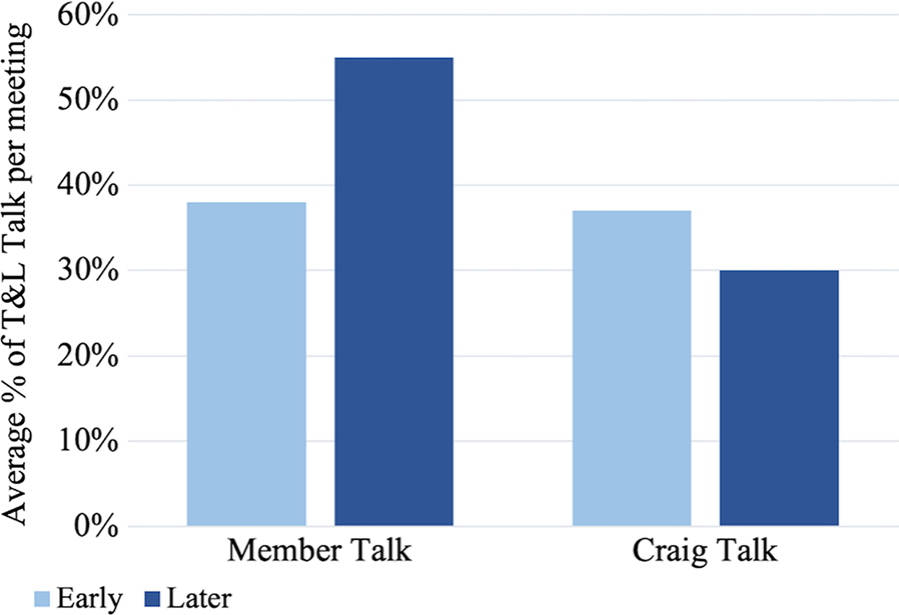
Average percentage of T&L talk duration of Craig and members during early and later group meetings. Early meetings averaged 60 min (averaging roughly 23 min of member talk and 22 min of Craig talk) and later meetings averaged 55 min (averaging roughly 30 min of member talk and 17 min of Craig talk). Co-facilitator talk is not represented on this graph

**Fig. 7:**
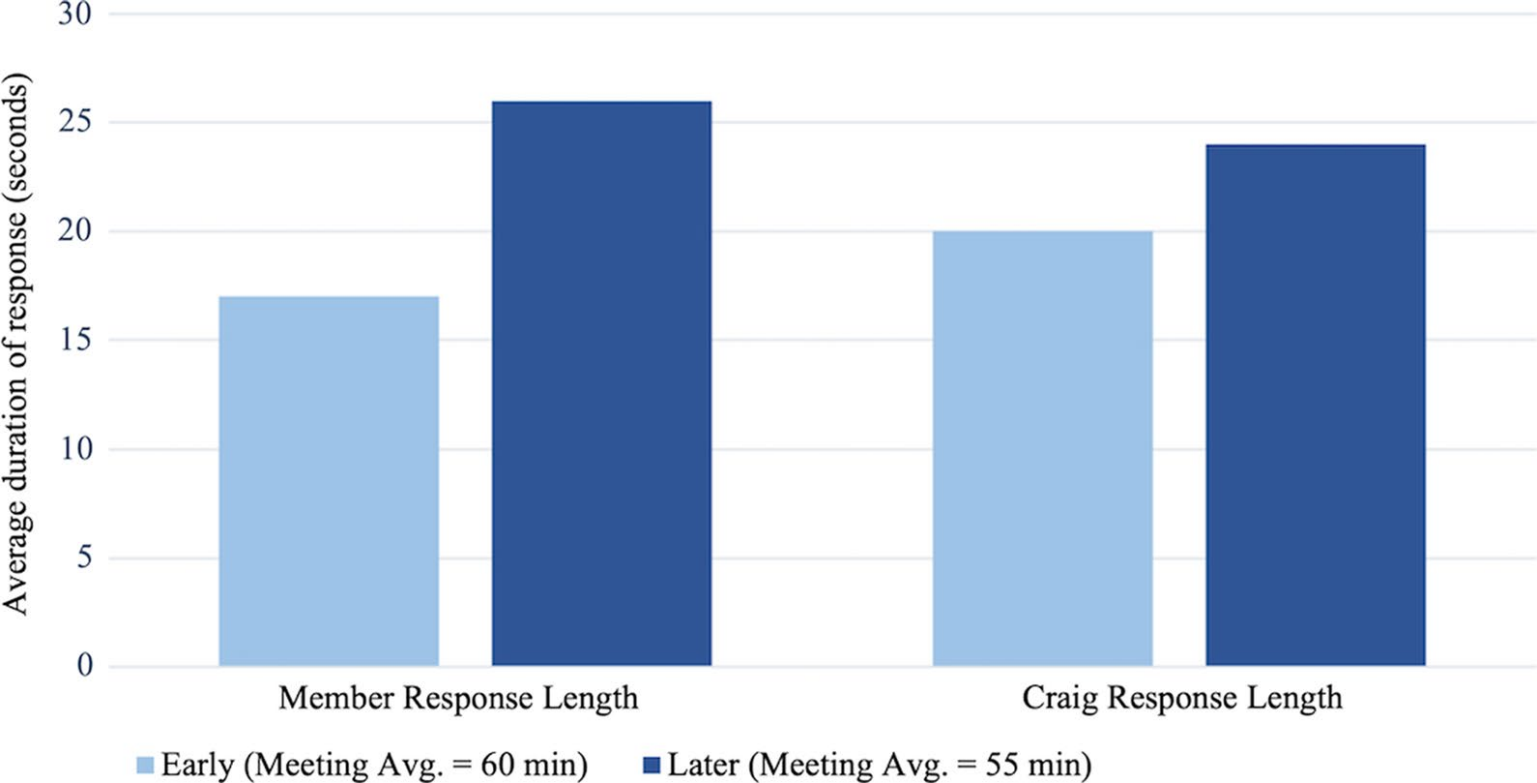
Average length of the responses of Craig and of members during early and later group meetings. Response duration is indicated in the average length (in seconds) of individual responses. Co-facilitator talk is not represented on this graph

Because Craig made more attempts to engage members in talk with more participant-focused responses, it would follow that members might speak more during later meetings in response to these attempts. To test this, we looked at the relative percentage of member and facilitator talk and the average duration of facilitator and member responses. Craig’s average contribution to the total T&L talk decreased, while members’ contributions to T&L talk increased in later meetings (Fig. 6). In early meetings, Craig and the members make up relatively similar percentages of talk, whereas in later meetings Craig’s talk decreased while the members’ increased. In addition, average talk duration (per response) for members increased (Fig. 7), meaning that members talked for a longer amount of time in each of their responses during later meetings. This suggests that as they gained experience, they had more to speak about regarding issues of teaching and learning, a point we return to when discussing the meeting content change theme below.

Overall, the data show compelling evidence for the change in Craig’s role from being the expert in the group focused on sharing expertise, to being more focused on drawing on members’ ideas and promoting their contributions to the discussion. These results support the role change theme and show that these efforts appeared to have been successful in promoting increased member contribution to discussions of teaching and learning.

### Response change

**Fig. 8:**
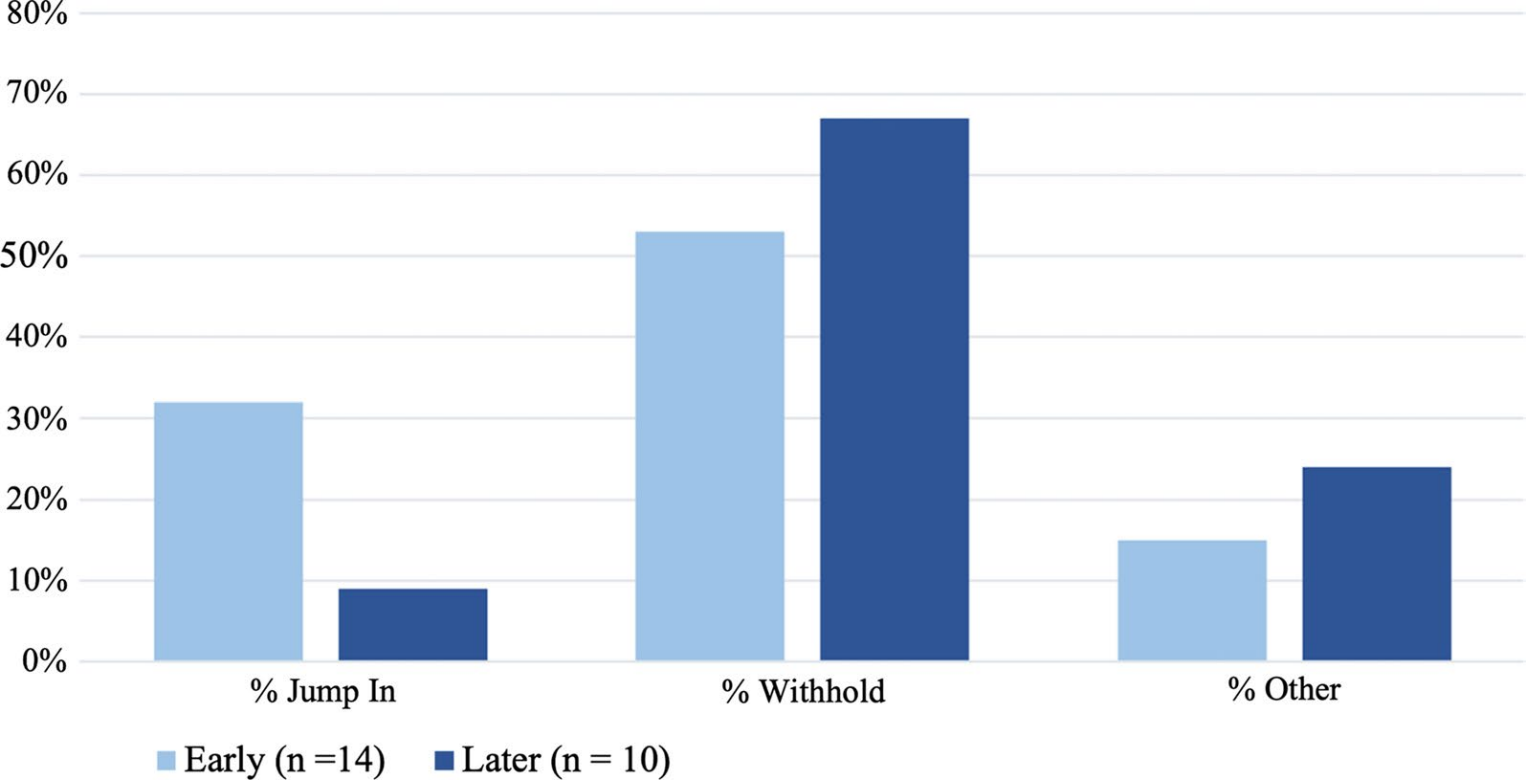
Results of segment sequence analysis. The percentage of segments in which Craig responded by *jumping* *in*, *withholding*, or providing another (*other*) type of response in early versus later meetings (*n* is the average number of segments in meetings during the five early and five later meetings)

In early meetings, Craig *jumped* *in* more often, and *withheld* less often compared to later meetings (Fig. 8), consistent with the response change theme. Additional evidence is the increase in the average number of *redirecting* *questions*, *revoicing* *comments,* *summarizing* *responses,* and *pedagogical* *questions* per meeting (Fig. 5), showing his increased effort towards types of responses that encourage other members to respond during discussion.

Other examples come from comments Craig made to group members during later meetings that minimize his role as the primary expert:

“I'm here to help lead and then also to learn just like everyone else.” [...] “So I'll be facilitating and then also paying attention at the same time because I have stuff to learn.” (9/20/19 FOLC group meeting)

“I'm all ears as well, because I'm getting ready to teach that [unit] for the first time in the Spring.” (10/18/19 FOLC group meeting)

“So, I liked this idea [...] but I don't want to talk too much [...]. [Are there] other topics or things that people have on their mind? [...] I want to make sure we all have a chance [to share]. [Are there] other logistical things that we need to talk about, textbooks, access to resources, that sort of thing? We're all pros at this now. So, we've got it all figured out, kind of.” (10/18/19 FOLC group meeting)

In later meetings, Craig positions himself to members as not wanting to take up talking time, empowers members as “pros”, and states his intention to learn from members himself. We did not find examples of this kind of talk in any of the early meetings. In a later facilitator meeting he even explicitly stated, “…if you have [group members] sharing out, that’s the thing that needs to be prioritized and be explored.” (9/20/19 Facilitator Video PD meeting) Altogether, these results support the role change theme.

### Meeting content change

**Fig. 9:**
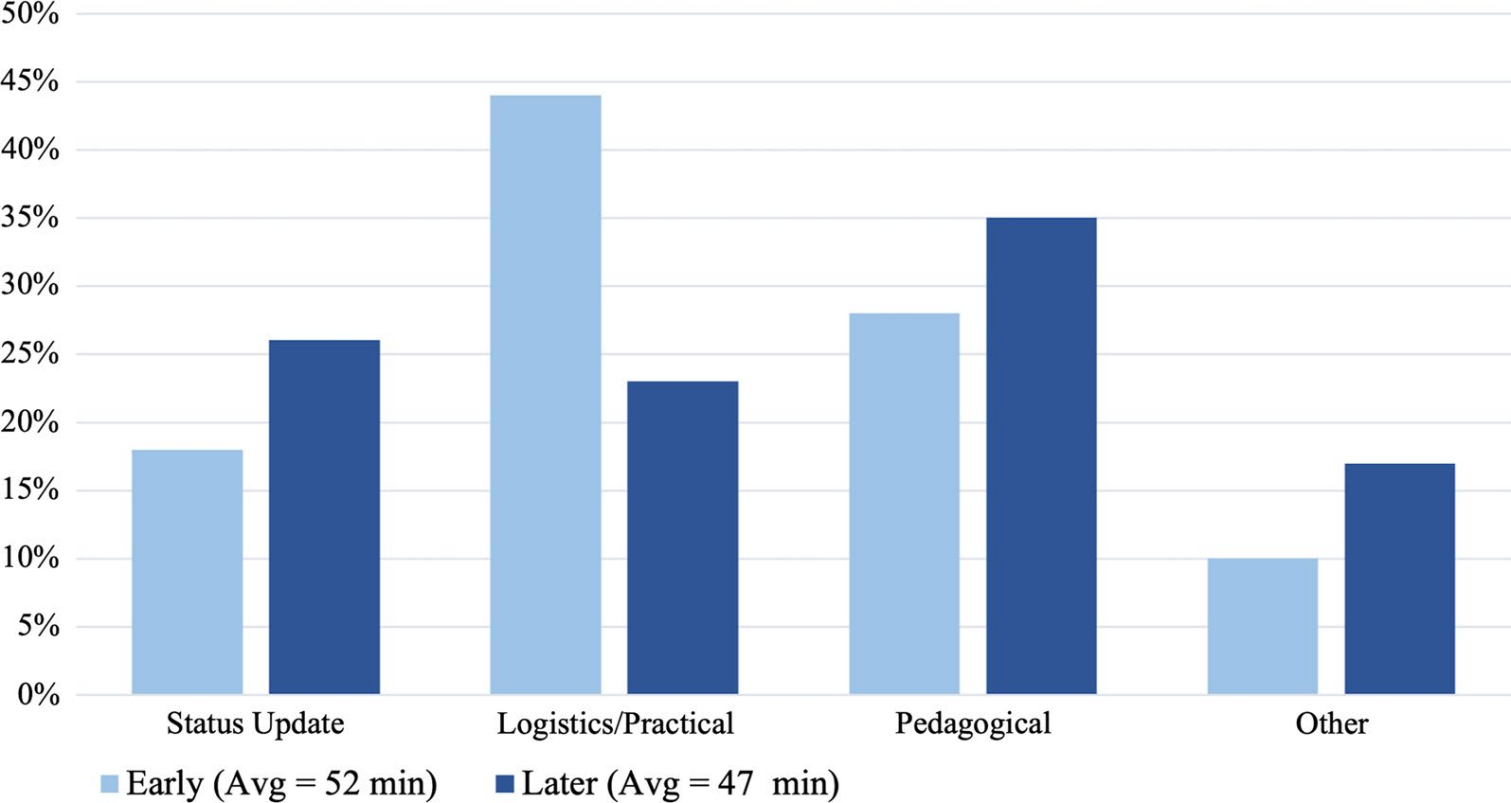
Duration of conversation coded for each Segment Content Category. Segment content categories coded are coded as relating to status updates, logistical/practical issues, pedagogical issues, or other kinds of discussions (as a percentage of the total T&L talk duration, shown in parentheses) in early versus later meetings

Early meetings had a longer average duration of logistical/practical talk and smaller duration of pedagogical talk. Comparatively, later meetings showed a decrease in the average duration of logistical/practical talk and an increase in pedagogical talk (Fig. 9).

These data support Craig’s observations throughout the project that new NGPET FOLC members tended to focus on logistics and practical issues:

“With the people who are just starting out, they’re very much in that, “What are the tips and tricks?” Like, “What do I need to know about this specific activity?” “What are the hidden needs that I'm going to run into and not be fully prepared for?” So, they're kind of logistical items that people are focused on.” (12/12/2018 Facilitator Meeting)

He believes that in the beginning, meetings were focused on addressing the “tips and tricks”, as desired by faculty new to the curriculum. However, later meetings were able to focus more on deeper (pedagogical) issues.

“I think there were a couple times where folks were [...] almost welcoming the deeper questions about: What does this mean for student learning? What does this mean for my teaching?” (12/11/2019 Facilitator Meeting)

He later notes that members were willing and able to discuss deeper issues of teaching and learning. Perhaps in response to these changing needs, or in an effort to promote more of these types of conversations, the meeting data revealed Craig also asks more pedagogical questions in later meetings (Fig. 5), for example:

“Do you have any further comment on *why* it is you think what you're doing is productive, *how* it's working?” (9/20/19 FOLC group meeting).

“[Are there] indicators or markers that you see happening in the class and based on [that], you know right then at that point that, ‘Okay, so we're not going to do this this semester and we're going to switch gears and do this *other* thing.’?” (10/18/19 FOLC group meeting)

“How does the curriculum transfer to what [students’] interests are? [...] Do you struggle with buy-in from students? “(3/02/20 FOLC group meeting)

In just these few examples, Craig asks *how* and *why* questions, explicitly asks faculty to reflect on their classroom experiences, and directs attention to student thinking. These kinds of questions, meant to draw out deeper conversation about teaching and learning, were less abundant in earlier meetings. Craig asked more pedagogical questions in later meetings (Fig. 5), and there was an increase in the average duration of pedagogical talk and a decrease of talk about logistics or practical issues (Fig. 9).

In summary, we found that our coding schemes and thematic analysis of a variety of data sources provided compelling evidence to support the claims described in all three change themes.

### Craig’s IMPG change sequence

**Fig. 10:**
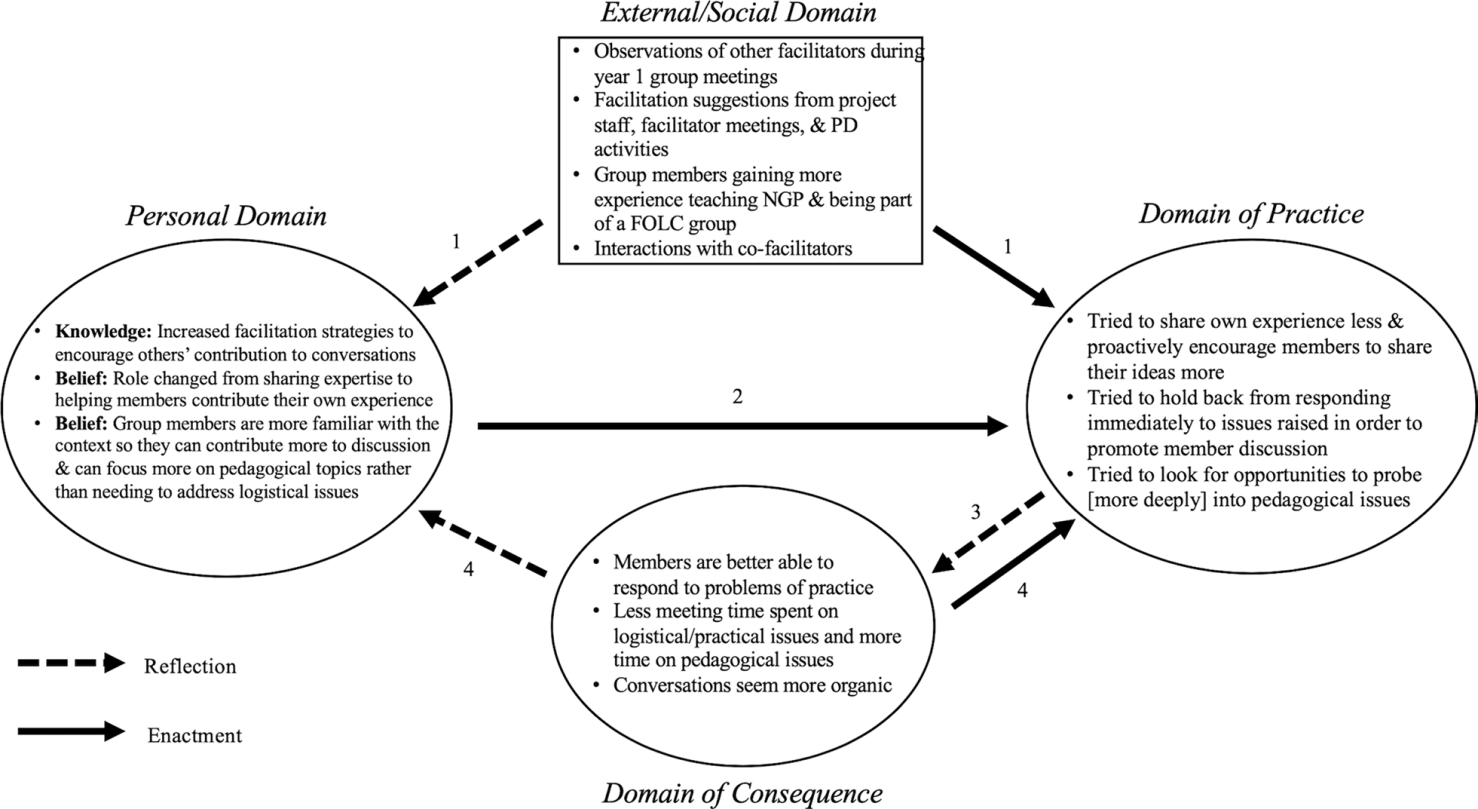
The IMPG change sequence for Craig as a facilitator over 2 years

We used the three change themes, our coding schemes, and thematic analysis of multiple data sources to construct an IMPG *change* *sequence*, presented below. As previously described, a change sequence is a connection between at least two domains of the IMPG, where information about the domain changes and mediating connections are based on empirical data. Our theme-based transcript analysis of ten FOLC group meetings, Craig’s ideas about his own change from the interviews, and Craig’s comments during facilitator meetings have informed the creation of a change sequence regarding Craig’s facilitation experiences (Fig. 10).

The evidence gathered from our investigation points to the *external/social* *domain* as the domain that initiated the influence on Craig’s changes as a facilitator. Here, the experience being a participant in a group for one year lead by other facilitators, interactions with the NGPET FOLC project team and other facilitators during meetings, facilitation suggestions, and guided co-facilitator planning/reflection meetings helped to develop Craig’s ideas about strategies to be an effective facilitator. These strategies included asking questions before giving advice, inviting other group members to weigh in on an issue, and seeking to compare/contrast ideas raised. Also, the video PD with the project staff helped develop facilitators’ skills by reviewing clips of different kinds of facilitation moves and encouraging facilitators to reflect on their practice. This video PD meeting seemed to have a particularly strong effect on Craig’s perception of his own role and strategies as a facilitator:

“So, looking at the transcript . . . it seems as though the [facilitator] . . . had a lot of voice, . . . occupying a lot of the time. And so that made me really reflect back on . . . my own experiences with the [group] last year, and it had me worried. It had me concerned, because one of the reasons we get into this business is we want to share our ideas, we want to be helpful. And the knee-jerk response or way to do that is to *provide* our help and to talk. And so, I really immediately felt concerned and was being critical of myself like, ‘Oh did I do this too much last year? Was I talking too much? Why didn't I redirect more? And why didn’t I let *them* make the comments and make the statements, and then draw the insights from what they had deep down?’” (9/19/2019 Facilitator Meeting)

After watching a video and reading a transcript of another facilitator in action, Craig realized that he may have been dominating the conversations in his first year as a facilitator and lamented that he did not draw on group members’ ideas. This realization may have also been promoted by another component of the *external/social* *domain*, the increase in members’ experience teaching the NGPET curriculum in his second year as a facilitator. Members had more knowledge of the curriculum to draw from going into their second (or later) years of implementation. Craig’s enactment of the suggested strategies and his reflection from the PD leads to two arrows in the IMPG: a dashed *reflection* *arrow* (1) to the personal domain and a solid *enactment* *arrow* (1) to the domain of practice (Fig. 10).

The reflection upon his experience as a member in year 1, his PD experience, FOLC group meetings, meetings with facilitators, and the increase in group member knowledge led to changes in his knowledge and beliefs about his role as a facilitator, represented in the *personal* *domain* (Fig. 10). Craig felt that one of the roles of the facilitators in year 1 was to help group members, “share ideas, … [and make] sure that everyone is contributing or feels as though they could contribute” (2018 interview). This impression likely influenced his ideas regarding his own role when he became a facilitator. Furthermore, the facilitator PD helped him develop practices to better enact this role of helping members share when he became a facilitator. He was introduced to strategies such as Horn and Little’s (2010) idea of *turning* *toward*: clarifying by asking “why” and “how” questions to understand the issue, and keeping agency with the problem poser before offering solutions or moving to a new topic. He also learned strategies by interacting with other facilitators and project staff during facilitation meetings:

“And I think it was just that with those [facilitation PD] suggestions for continuing the conversations. . . . I don't think these words were used, but basically it encouraged us to try to “bite our tongue” a little bit. Let *them* have the discussion. And I felt myself really wanting to make a point or share, and biting my tongue letting them talk. And then the door would open and it's like, ‘Okay well I just can't hold back anymore.’ I want to share a little bit about some experience . . . [I] think what was really helpful for me was, yes, bite your tongue as the facilitator, and then pull it out of *them*. You know, pull it out of *them* what it is that's going on. So, I felt for me there was a lot of growth there.” (12/11/2019 Facilitator meeting)

The facilitation suggestions provided in the beginning of fall 2019 (Fig. 2; full suggestions provided in Additional file 1) had advice that Craig interpreted as suggesting that facilitators “bite their tongues” to draw on others’ ideas. His increase in knowledge about how to draw on members’ ideas and reflection on these suggestions enabled him to change his beliefs about his role. Craig felt he no longer served as a facilitator entirely to share his own experience, but to hold back and help “pull” ideas out of the group members, “talking about [the facilitator’s] personal experiences, […] is also important, but if you have people sharing out, that’s the thing that needs to be prioritized and be explored.” (9/19/2019 Facilitator meeting).

These changes in his knowledge and beliefs about facilitation were also enacted (*enactment* *arrow* 2 in Fig. 10) in changes in his facilitation strategies represented in the *domain* *of* *practice*, as documented in our analyses of FOLC group meetings. Craig decreased his *facilitator* *focused* responses (Fig. 10) sharing his own *experience* and *solutions* less frequently, while showing an increase *participant* *focused* responses, using more *redirecting* *questions*, *revoicing* *comments*, and *summarizing* *responses*, to draw out or build upon members’ ideas (Fig. 5). In his interview, Craig describes his hope for members to share and develop on each other’s ideas, and how he attempts to promote this by “unpacking” members’ ideas and “bouncing around” to others in the group (Table 3; see Additional file 6: Table S1 for additional supporting quotes). Another change in strategy represented in the *domain* *of* *practice* is Craig’s attempt to try and hold back his own response to promote member sharing. In later meetings Craig *jumped* *in* less frequently to give advice or answer questions and increasingly *withheld* his response or redirected to others (Fig. 8).

These changes in practice were apparently successful, as group members increased their proportion of teaching & learning (T&L) talk (Fig. 6). Average response length for members also increased, possibly indicating that they were sharing more substantively (Fig. 7). Craig also seemed to successfully hold back, his duration of T&L talk decreased in later meetings (Fig. 6), stemming from his reflection upon his facilitation strategies. In the prior quote from the facilitator video PD meeting, Craig questioned if he was sharing too much during FOLC group meetings. In doing so, he established a connection (*reflection* *arrow* 3, Fig. 10) between the *domain* *of* *practice* and the *domain* *of* *consequence*.

Craig noticed a few salient outcomes about the groups he had been facilitating over time. He found that the barriers between facilitators and group members were starting to dissolve, with members taking more initiative in posing and responding to problems of practice. Craig observed in his interview that, while initially he was a mentor, later there was less of a hierarchy and everyone was mentoring each other and sharing ideas (Table 3; see Additional file 6: Table S1 for additional supporting quotes). This, in turn, impacted how conversations unfolded in the group. Because of the growing experiences of the group, he noted that the nature of the conversations become more organic (Table 3; see Additional file 6: Table S1 for additional supporting quotes). In addition, the facilitation guidelines handout, and the opportunity to interact with co-facilitators before and after meetings (which started in the second semester of 2019–2020) seemed to enhance the ability to foster more organic conversations.

**“**I like [the co-facilitators] meeting beforehand a little bit just to get in the mindset. I'm not the most gregarious or I don't have the gift of gab. So, I appreciate you [co-facilitator] chiming in when things fall flat. . . . Sometimes I think I'm guilty of trying to force the meaningful conversations instead of letting them come out organically, so I'm working on that. I guess one strategy is not to have the meeting planned out to the nth degree, but we should probably . . . take a look at the suggested list of prompts and maybe pull from that a little bit more or have them in our back pocket at least.” (3/02/20 Post FOLC group meeting co-facilitator reflection)

Craig notes that meeting with his co-facilitator in advance is helpful to “get in the mindset” and reflects that he is trying to let conversations happen more organically by not fully planning out meetings in advance, but instead possibly utilizing the prompts provided by the research team (see Facilitation Guidelines in Additional file 1) to keep the meaningful conversations going. Through Craig’s influence, their growing experience in the FOLC group, their experience with the curriculum, or the nature of the more organic conversations, Craig noticed that members were more engaged with discussions of a pedagogical nature:

“. . . last year . . . with such new people where I was used to engaging with people about these logistical items. This go-around it was a little bit above that”. . . . “I think there were a couple times where folks were . . . almost welcoming the deeper questions about: What does this mean for student learning? What does this mean for my teaching? And so those were nice.” (12/11/2019 Facilitator Meeting)

In his 2018 interview, he noted that during the previous year the group had focused on practical and logistical issues rather than deeper pedagogical issues, addressing “... topics [that] were generally pretty fine-grained and... dealing with either very specific items of the curriculum or the logistics of implementing” (2018 interview). This may have influenced his expectation that in his first year of facilitation, members were going to be similarly focused on discussing issues related to logistics. However, as described in the quote above, in his second year of facilitating the members were more able and willing to discuss deeper topics of teaching and learning. This transition from a focus on smaller-scale logistical problems to a focus on larger-scale pedagogical issues is consistent with the Concerns-Based Model of Adoption (CBAM; Anderson, 1997).

His observations of conversations being less logistical and more pedagogical in nature were documented in our *segment* *content* analysis, with a decrease in talk focused on logistics or practical issues and an increase in pedagogical talk (Fig. 8). This reflection bolstered his belief (*reflection* *arrow* 4 from the d*omain* *of* *consequence* to the *personal* *domain,* Fig. 10) about members being capable of contributing their own experiences to enhance conversations and the ability of the group to discuss deeper pedagogical issues. He also acted upon these outcomes (*enactment* *arrow* 4 from the *domain* *of* *consequence* to the *domain* *of* *practice,* Fig. 10) by trying to draw upon more pedagogical ideas. During his interview, Craig expressed his desire for meetings to help faculty increase the scope of their discussions to improve the curriculum and their own teaching (Table 3; see Additional file 6: Table S1 for additional supporting quotes). Craig also seemed to try to promote these discussions by asking an increased the number of pedagogical questions (Fig. 5).

Analysis of the FOLC group meetings and review of multiple data sources (including the thematic analysis of meeting participation and the three coding schemes: *participant* *vs.* *facilitator* *focused* coding scheme, *segment* *sequence* coding scheme, and *segment* *content* *coding* *scheme*) show the three change themes identified in Craig’s 2020 interview appear to be supported by empirical data. The IMPG framework allowed us to construct a narrative of how these changes may have taken place, giving us a richer understanding of how these changes were supported. Consequently, Craig's IMPG diagram (Fig. 10) and its corresponding explanation, answers our research questions:

**RQ1**: How do a FOLC facilitator’s goals and strategies change over multiple semesters in the FOLC?

**RQ2**: What factors seem to influence the changes in the facilitator goals and strategies?

### Implications for community-based professional development

Although one needs to be very careful in generalizing from a single case, we believe there are lessons from Craig’s experience that could be useful to other facilitators or community leadership. This study may provide useful ideas about how to prepare and provide on-going support for facilitators of FLCs or FOLCs with the following characteristics: (1) the goals include supporting faculty who are starting to implement a RBIS; (2) member participation extends over a sufficiently long time period (e.g., two or more semesters) so members not only become skilled in implementation, but also seek to understand the impact of the RBIS on student learning and their own teaching; (3) facilitators have prior experience implementing the RBIS, but do not necessarily have prior experience as FLC or FOLC facilitators—that is, the facilitators learn and enhance their facilitation skills with experience; and (4) community leadership can provide opportunities to help facilitators enhance their craft and become more reflective practitioners.

The implications for FLC/FOLC facilitators gleaned from this study include the following:

- Facilitators may expect that logistical/practical issues will need to be addressed early on, and discussion of pedagogical issues will become more important as members gain experience. More generally, the needs and understandings of group members will evolve over time and developing a sensitivity to these changes is an important aspect of being a responsive facilitator.
- Facilitators may find it useful to monitor the participation of group members to ensure all are being given opportunities to raise issues and share ideas.
- Facilitators can monitor their own role to ensure that as other group members become more experienced, they withhold sharing their own experience and suggestions too soon in the conversation.
- Community leadership could provide opportunities to help facilitators reflect on and enhance their practice, including: selecting facilitators who are not only experienced in implementing the RBIS, but seem to be reflective practitioners; organizing periodic meetings of facilitators to share challenges and strategies to address them; using videos and/or transcripts of FLC or FOLC group meetings to discuss facilitation actions and inferred strategies; distributing facilitation suggestions from the research literature or from previous implementations of the FLC or FOLC (see Additional file 1 for some suggestions); and using structured prompts to help facilitators plan for and reflect on their group meetings (see Additional file 2 for some suggestions).

For science education researchers who study the professional development of facilitators, we have two methodological suggestions from our case study. First, we find that the IMPG (Clarke & Hollingsworth, 2002) with the modified *External/Social* *Domain* provides a useful and substantive framework to study facilitator change. Second, some or all the coding schemes we developed for our analysis of Craig’s changes might be useful analytical tools if the study of facilitators takes place in FLCs or FOLCs that incorporate the facilitation suggestions listed above. Although the themes in our study were tailored to Craig, at least the role change theme and meeting content change themes are like the changes that would be expected in the context of professional growth, based on the Concerns-Based Adoption Model (Anderson, 1997). This suggests that the coding methodology described in this study may be of use to future investigations of change in other contexts of professional growth.

### Limitations

One limitation of this study is the fact that (nearly) all the FOLC group meetings were co-facilitated. This means that, although we focused on analyzing Craig's actions and responses, we cannot entirely disentangle the effects of his behavior from those of his co-facilitators in our findings. Also, while we analyzed all the meetings that fit within our selection criteria (Craig and three or more group members present), we analyzed only 10 out of 28 meetings that Craig facilitated or co-facilitated over 2 years. We do not know how our findings might extend to meetings where attendance was limited to fewer than three group members. Finally, we acknowledge that the composition of his groups during later meetings (each semester) were different than during his early meetings, and therefore might have had an impact on the differences observed between early and later meetings.

## Conclusion

In this article, we presented a case study of Craig, a facilitator in the NGPET FOLC, who changed his facilitation goals and strategies in response to both PD efforts and to changes in his group members’ curriculum expertise and experience in the FOLC. We provided empirical evidence from interviews, facilitator meetings, and analyses of the FOLC group meetings he facilitated over the course of 2 years, to construct an IMPG diagram (Fig. 10) for Craig. The diagram served as a pictorial depiction to answer our two research questions: (1) How do a FOLC facilitator’s goals and strategies change over multiple semesters in the FOLC? With answers provided in the *Domain* *of* *Practice* and the *Personal* *Domain*. (2) What factors seem to influence the changes in the facilitator goals and strategies? With answers provided in the *External/Social* *Domain*, the *Domain* *of* *Consequence*, and the enactment and reflection arrows that connect the four domains.

Our findings may have implications for other FLCs and FOLCs. Awareness of both the *enactment* and *reflection* mechanisms can guide community leaders to design specific activities to help foster change. Despite our case study focus on a single facilitator, we suggested some strategies that facilitators could productively attend to, discussed in the *Implications* *for* *Community-Based* *Professional* *Development* subsection. The suggestions involve being prepared for the changing needs of group members over time (Anderson, 1997), monitoring of member participation, and self-monitoring of facilitators’ response to member’s issues. Furthermore, community leadership can help promote facilitator change by providing opportunities to reflect on practice, as this was found to be an important mechanism to foster the kinds of changes that could help lead to productive group conversations and better support community goals.

## Supplementary Information

**Additional file 1**. Facilitation suggestions.

**Additional file 2**. Planning/Reflection suggestions.

**Additional file 3**. Interview protocol.

**Additional file 4**. Transcript segmentation & preliminary analysis.

**Additional file 5**. Case facilitator interview transcript.

**Additional file 6**. Supporting quotes for three change themes.

## Data Availability

Additional files contain some of the primary data sources, including the full transcript of the 2020 interview with the case facilitator. Aside from the interview, primary sources, such as meeting transcripts, are not available to the public.Makenna M. Martin is a PhD student in the SDSU and UCSD math and science education joint doctoral program. Fred Goldberg^1^ is an emeritus professor of physics at San Diego State University and member of the Center for Research in Mathematics and Science Education. Mike McKean is a member of the Center for Research in Mathematics and Science Education at San Diego State University. Edward Price^2^ is a professor of physics at California State University San Marcos and director of the Center for Research and Engagement in STEM Education. Chandra Turpen^3^ is a Research Assistant Professor in the Department of Physics at the University of Maryland, College Park, specializing in Physics and Engineering Education Research.

## Abbreviations

STEM: Science, technology, engineering and math
FLC: Faculty Learning Community
FOLC: Faculty Online Learning Community
RBIS: Research-Based Instructional Strategies
CBAM: Concerns-Based Adoption Model
NGPET: Next-Generation Physical Science and Everyday Thinking
IMPG: Interconnected Model of Professional Growth
PD: Professional development
