# Supplementary material for: Understanding how facilitators adapt to needs of STEM faculty in online learning communities: a case study
Source: Int J STEM Educ. 2022 Sep 5;9(1):56. doi: 10.1186/s40594-022-00371-x (PMC9443628; doi:10.1186/s40594-022-00371-x)
Supplement: Supplementary file 1 — Additional file 1. Facilitation suggestions. [file 40594_2022_371_MOESM1_ESM.docx]

**Supplemental Material: Facilitation Suggestions**

[These suggestions were emailed to facilitators in August 2019 to provide some suggestions for discussion strategies and giving some example conversation topics]

Background and goals:

By now, most FOLC members have taught NGPET more than once. Logistical and management issues may still crop up and need to be addressed, but we expect this to be significantly reduced compared to before. As a result, FOLC meetings can push to promote deeper pedagogical understanding and substantive opportunities to learn.

What to talk about?

- Start with a very abbreviated round robin: “Is there an issue you’d like to have more conversation about?”
- Note issues likely to be of most interest to most people, and to drive the deepest discussion, and follow up on that first.
  - You don’t have to get to everything.
  - Some issues lend themselves well to discussion/resolution on Slack - especially practical/logistical questions or sharing resources.
- Have a backup plan/topic for discussion (some possibilities are listed at the end of this document).
- Consider taking notes in a slack thread - and particularly noting things for follow up, such as implementation changes that faculty consider.

Promoting engaging discussion and opportunities to learn:

*Turning towards teaching*. The idea is to dig into problems to understand them, generalize, and ultimately gain insight into teaching and learning; in contrast to providing a quick fix or set of suggestions. Horn and Little describe turning towards teaching: “[treat] the shared and expected (normal) character of a problem as the starting point for detailed discussion of specific classroom instances and as a means to help anchor emergent advice to more general problems and principles of teaching. In these interactions, the problem teller was positioned with substantial agency in defining and elaborating on the problem and in working out possible responses.” Horn and Little (2010).

*Routines for turning towards*

Ask questions, clarify the issue, and seek to understand the situation **before** offering solutions.

Draw out more details about classroom events that unfolded to support building inferences.

Get poser to *explain the why* behind the problem. How have you tried to address the issue, and *why* do you think it worked (or didn’t)?

Try multiple framings of the problem’s nature and possible causes

Where does the problem originate (Curriculum? Students? Faculty? A combination?)

Look to others’ experience - has anyone had a similar issue?

Returning to the problem poser - is the discussion still addressing their issue?

Generalize and connect to more formal concepts - what is common/similar in our experiences? Are there any concepts to help us understand this?

*Some specific suggestions:*

- When a member describes an issue/problem, encourage others in the group to participate in the discussion (e.g. sharing similar or related experiences, helping to craft solutions).
- When more than one idea or perspective is presented to address a problem or issue, encourage comparing/contrasting the ideas or developing them further. One prompt that may help is to ask ‘what’s your pie in the sky [ideal] solution/outcome?’
- When a member describes a pedagogical problem, encourage him/her to provide a pedagogical explanation for why it is a problem.
- When a member generates a solution to a problem, encourage him/her to also provide a pedagogical explanation for why it is a problem.
- When a member includes his//her own classroom experiences when describing a pedagogical problem, encourage him/her or others in the group to make explicit connections to general principles of teaching and learning.

**Discussion topics**

Ideally, member concerns, questions, and ideas will drive the discussion during FOLC meetings. Even seemingly superficial topics or questions can often lead to substantive discussion if the group chases the why behind the issue, connects to other experiences, and seeks to generalize their understanding. However, if all else fails, these topics can be used to spark discussion.

Backup topics for generative discussion: Use these to promote deeper discussions if FOLC members do not have driving questions for the meeting. Probe for evidence and rationale/explanation for why it could/would work.

- How do you set norms at the start of the term?
- How do you deal with groups working at different paces?
- What do you do when activities do not take the entire class time?
- How do you encourage and monitor participation by all members within a group?
- How do you generate engaging whole-class discussions? How do you encourage all students to participate?
- How do you make students comfortable sharing their ideas with you? With the whole class? What if they want to know they are right before they present to the whole class?
- Do you do any sort of review at the start of class? How do you do it? How does it fit with the pedagogy of the course?
- How do you construct exams? Short answer/MC/mix? How do students get feedback to prepare for the short answer questions? How do you handle grading them?
- Do you offer group exams or portions of exams that involve group work? If so, how do you handle that?
- Do you have student assistants to help with the class? How do you prepare your TAs/get them on board for this type of pedagogy?
- What have you learned from your students since the last call (any courses)?
- What lessons from other courses can be helpful in NGPET or vice versa?
- What ideas do you have for supplementary activities or homework assignments?
  - What is one change that you've made to the curriculum?
  - [requires some prep in advance] Have people bring in exam/test questions they have written, their goal for the question, how well the question worked, and how it provided a window into student thinking
  - [requires some prep in advance] Share and discuss examples of student work.
