# Supplementary material for: Understanding how facilitators adapt to needs of STEM faculty in online learning communities: a case study
Source: Int J STEM Educ. 2022 Sep 5;9(1):56. doi: 10.1186/s40594-022-00371-x (PMC9443628; doi:10.1186/s40594-022-00371-x)
Supplement: Supplementary file 2 — Additional file 2. Planning/Reflection suggestions. [file 40594_2022_371_MOESM2_ESM.docx]

**Supplemental Material: Planning/Reflection Suggestions**

[Emailed to facilitators in January 2020]

To help us learn more about the facilitator’s role in FOLC meetings:

- We are asking that you meet your co-facilitator online shortly before & after each of your group’s Zoom meetings.
- We have provided planning and reflection questions to guide your discussion.

The NGPET FOLC project team is working on developing a facilitators’ guide that could be useful to those planning their own online learning community. The project research team has also begun investigating how facilitator prompts can influence the nature of discussions that occur in the FOLC meetings. To provide insights for both the facilitator guide and the research effort we have developed a new protocol aimed at helping facilitators in planning and reflecting on meetings. It consists of a short list of questions that you and your co-facilitator can think about and discuss before and after each meeting; goal-setting/planning pre-meeting and reflection post-meeting. We anticipate these conversations will be brief, and not take up too much of your time, while providing a benefit to both yourself and the community. Literature on facilitation suggests that reflection may be helpful in enhancing facilitation skills. As well, these facilitation reflections will help to identify key aspects of practice that contribute to productive discussions in online learning communities, helping us to generate “best practices” for new FOLC facilitators.

Logistics: Please plan to meet with your co-facilitator 5-10 minutes prior to, and at the end of each meeting on Zoom. Prior to each meeting you will discuss your goals for the meeting, and following the meeting you will be discussing how the meeting went. In order to help our research team develop recommendations for effective FOLC facilitation strategies and their relationships to the nature of the discussions during the meetings, your short pre- and post- meeting discussions will be monitored. By monitoring the video we hope to minimize the time burden of this activity on you; we can glean insights into the FOLC community without you needing to write down answers or coordinate a separate meeting. You may recall the facilitation suggestions provided last semester, these also may be helpful as you plan your meeting goals.

Pre-Meeting Planning: On Zoom, we suggest discussing one or more of the following topics with your co-facilitator prior to when the other members show up.

1. What are some of your goals for facilitating this session?
   - e.g. aspects of your facilitation, what you hope members will take away from the meeting, any topics from a prior meeting that you hope to cover this time, etc.
2. Are you planning to fulfil specific roles for this meeting?
   - e.g. one person lead and the other takes notes, both lead, etc.
3. How do you plan to structure the meeting?
   - e.g. round robin for problems and then discussion, etc.

Post-Meeting Reflection Topics: On Zoom, please reflect on questions 1, 2, and at least one topic from 3 with your co-facilitator after the other members leave the meeting.

- To what extent did you accomplish your goals for this meeting?
  - Why or why not?
- Did the co-facilitator roles play out in this session the way you planned?
- How do you think the session went? More specifically, you could address one or more of the following:
  - Were there interesting and/or surprising things in this session?
    - What were they?
- Why were they interesting or surprising?
  - Were there any challenges that you encountered in this session?
- e.g., challenges in facilitation, discussion, group dynamics, etc.
- What were they?
- How might we address these going forward?
  - Did you feel the agency within the group shift (either from you to the group, or from the group to you)?
    - When?
    - What did you feel and do at that moment?
    - Why do you think this happened?
  - Were there moments when you pressed the group (and yourself) to stick to your original facilitation plan rather than allowing the group to make a spontaneous shift, or vice versa?
    - What did you base the decision on?
    - What were the results?

In order to help us understand the nature of FOLC meetings, please try to anchor your responses in the specifics of the meeting that just took place.
