# Supplementary material for: Understanding how facilitators adapt to needs of STEM faculty in online learning communities: a case study
Source: Int J STEM Educ. 2022 Sep 5;9(1):56. doi: 10.1186/s40594-022-00371-x (PMC9443628; doi:10.1186/s40594-022-00371-x)
Supplement: Supplementary file 3 — Additional file 3. Interview protocol. [file 40594_2022_371_MOESM3_ESM.docx]

**Supplemental Material: Interview Protocol**

[Interviews conducted with FOLC facilitators in Fall of 2020]

1. What is your role as a facilitator? [Clarification - role: the social part they play in the conversation] Please explain (give examples if appropriate).
   - How do you think you’ve accomplished/enacted this role?
   - Do you think your role has changed over the duration of the project? Why did it change?
   - What was your experience with facilitation prior to your involvement with this project?
2. What are your facilitator goals during this project? [Clarification - goal: what they intend/hope will be accomplished - outcomes] Please explain (give examples if appropriate).
   - Have your facilitator goals changed over the 2-year duration of the project? How and why?
   - If they say something about productive/good/learning (USE THEIR TERM) discussions: What do you see as characteristics of a productive/good/learning conversation?
     - Has that view changed over the course of your involvement with the project?
   - Did you feel like your conversations were more centered on giving solutions and solving things immediately (logistics/solutions) or talking more about the pedagogical implications and the deeper meaning behind things?
     - How has this changed over the course of the project?
3. What actions or strategies did you use to achieve your goals? Probe for some specific examples.
   - Ask about any approaches or strategies they found to be particularly effective (for their goals, for generating discussion, for encouraging participation, etc.).
4. How personally successful do you think you were in achieving your facilitation goals?
   - Where do you see yourself as having been successful?
   - What has been challenging as a facilitator?
5. How did your co-facilitator and/or group members help you to achieve your goals?
   - How did you share responsibility with your co-facilitator for achieving your goals? How do you think that worked out?
   - What role did you see the group members play in helping achieve your goals? Can you give some examples?
6. Over the course of the project, what has helped you become a more effective facilitator? Probe for some examples.

- How did the discussions with other members and facilitation guidance from the project team affect your role as a facilitator or how you implemented it (if at all)?

1. Over the next year we are going to be developing a guide for new facilitators. Based on your experience as a facilitator over these past few years, what advice would you give to new facilitators?
   - What could the project team do to better support facilitators?
   - How do you think these facilitated meetings have been helpful for developing members’ skills related to teaching and learning?
2. How might you do things differently in the future? Why?
3. Have you applied what you have learned as a FOLC facilitator to other facilitation contexts?
