# Supplementary material for: Understanding how facilitators adapt to needs of STEM faculty in online learning communities: a case study
Source: Int J STEM Educ. 2022 Sep 5;9(1):56. doi: 10.1186/s40594-022-00371-x (PMC9443628; doi:10.1186/s40594-022-00371-x)
Supplement: Supplementary file 4 — Additional file 4. Transcript segmentation & preliminary analysis. [file 40594_2022_371_MOESM4_ESM.docx]

**Supplemental Material: Transcript Segmentation & Preliminary Analysis**

[Process of segmenting transcripts for analysis]

Goal of Transcript Segmentation & Preliminary Analysis: Capture the structure and flow of conversation during a NGPET FOLC meeting, with particular attention to the moves (questions, discussion topics, sequences of interaction) from both facilitators and group members that initiate discussion around issues of teaching and learning (T&L). In this codebook, we describe how we code a transcript.

**Identifying T&L Segments and Prompts**

Teaching & Learning (T&L) segments**:** For each meeting you read through the transcript and demarcate the conversation into segments by topical shifts, each beginning with a prompt posed as a question or topic focusing on some issue regarding teaching and learning, and that generates multiple (two or more) responses, or one substantive response, from group members. These segments are called Teaching and Learning (T&L) segments.

- They are distinguished from other conversations within the meeting that focus on other issues that are backgrounded in the analysis and are not coded; e.g., issues of a personal nature, how the FOLC works, or social chit-chat. These other issues may serve an important function for the operation and health of the FOLC, but are not focused specifically on issues of teaching and learning.

Prompts**:** There are two types of prompts: Question prompts and discussion Topic prompts.

- Question (Q) prompts are intentional in the sense that the person posing the question is seeking responses from other group members. Most question prompts are actual questions, but sometimes a person will frame a request for responses or feedback without asking a question explicitly.
- Discussion Topic (T) prompts are unintentional in the sense that the person discussing some topic is not explicitly seeking responses or feedback from others. However, the topic mentioned by a group member is only coded as a discussion topic prompt if it generates follow-up discussion from other group members.
- There are two categories of participants at meetings: facilitators and non-facilitators.
  - Prompts can be posed by either category of participant, facilitator or non-facilitator.
- The T&L Flow of Conversation during a meeting consists of the sequence of T&L segments, each consisting of its associated prompts and responses.
- In some meetings a facilitator poses the same prompt question multiple times during the meeting to encourage several participants to respond to the same question. Because we are mainly interested in unique prompts, our code labeling scheme does not differentiate between multiple instances of the same question prompt being posed. However, the contribution type can indicate that this prompt is being posed again.
- If, during a segment, a person asks a clarification question (e.g. “Can you explain that more?”), that will *not* be coded as a new prompt, initiating its own T&L segment.

**Coding responses:**

- The person who poses the prompt can also respond to it.
- In order for a question prompt to be identified and coded, it must be followed by either a substantive response from one different person or two or more responses. If that condition is not met, that segment would not be coded separately.
  - For example, if Peter asks a question and only one person responds, “I agree with that,” then Peter’s questions would not be coded because it really doesn’t initiate a substantive discussion.
- The response section of a T&L segment usually includes several turns of talk between the prompt poser and two or more other group members, or between other group members. Generally, a participant will be coded as providing a response only once during that segment, unless he or she provides two or more responses that are significantly different from each other, and are separated by at least one other turn of talk. In that case, each significantly different response from the same person will be coded as a separate response/ On the other hand, if the poser and respondent engage in several turns of talk where the respondent extends or clarifies his/her original statement, that would be coded as one response from the respondent.
- Although most T&L segments are continuous, there are some that are discontinuous; i.e., the initial probe is offered at one point in the meeting and some responses occur later in the meeting (either spontaneously or with an identical probe). In those cases, the response is coded as belonging to the original prompt.
- Not all utterances by group members following a posed prompt (question or discussion topic) would be coded as responses; e.g., if a participant only makes a cursory, non-substantive, comment, following a question or discussion topic, that participant’s comment would not be coded as a response to the prompt.
