# Supplementary material for: Understanding how facilitators adapt to needs of STEM faculty in online learning communities: a case study
Source: Int J STEM Educ. 2022 Sep 5;9(1):56. doi: 10.1186/s40594-022-00371-x (PMC9443628; doi:10.1186/s40594-022-00371-x)
Supplement: Supplementary file 5 — Additional file 5. Case facilitator interview transcript. [file 40594_2022_371_MOESM5_ESM.docx]

**Supplemental Material: Case Facilitator Interview Transcript**

[Bolded text in transcript corresponds to the quotes included in Supplementary Table 1]

| Time | Speaker | Transcript |
| --- | --- | --- |
| 0:03 | Interviewer | Okay. So I just want to confirm that you understand that you're participating in a research study, that you understand that this is being recorded and that you've had a chance to ask any questions and get your questions answered and that you're in agreement to participate. |
| 0:21 | Craig | Yes, I understand. |
| 0:22 | Interviewer | Excellent. Thank you. |
| 0:25 | Craig | And give my consent. |
| 0:26 | Interviewer | Yes. All right. That's what I need. Thanks. So to start out, I have sort of two questions that are related and let me explain them both and then I'll go back to the first one. I'm interested in what you see your role is as a facilitator and also what your goals are in terms of outcomes. And so, role would be more like how you show up socially, what your role is in what takes place. And then the goals are sort of the objectives of what you hope gets accomplished. And so let's start with your role. How would you describe your role as a facilitator in the Next Gen FOLC? |
| 1:09 | Craig | I see my role as being more of a mentor to other faculty who are teaching the same course. I mean, that's what links us together is that we all teach the same course. But what I found is that my role as a mentor, first and foremost, is for the curriculum for Next Gen PET. But also I think, it's important and what's come out of participating is that we serve as mentors to each other in general. So we first tackle Next Gen PET issues, pedagogies, concerns, that sort of thing. But then also people draw on their experiences from other courses and I do as well. I think... And this might be a topic for later on, but if there's evidence, I think that a lot of what happens and what has happened in Next Gen PET has spilled over into other teaching, our other practices in other courses. So getting back to the original question, **I see my primary role as being a mentor, just helping organize discussions, lead the discussions to some extent, but really when we first started, it was we very much lead and then, as we kind of continued with the program or the project, it was, "How do we get the others, the other people who are in the meeting to help lead the discussion?"** [Table 3, Quote 6] |
| 2:40 | Interviewer | Okay. And has the way that you've viewed your role changed over time? |
| 2:50 | Craig | So good question. I guess, I'm talking now thinking about where I am now. Yeah, I think it has, I'm thinking back, **reflecting back and kind of retrospectively now and seeing myself as serving as a mentor, but also others are mentoring me as well. It's kind of like this, it's much less explicitly directed by one person who's leading everything, leading the charge. And now it's more like we're trying to get a conversation and a dialogue going, where it's not just the leader that has things that he or she, or they need to share. It's everyone collectively**. [Table 3, Quote 1] I**nitially I think it was very nuts and bolts and logistical. Our job was to get in touch with the other faculty who were teaching the curriculum and identify the issues, identify what the immediate needs were.** And this could be very technical like Unit M, activity four, question three, what's meant by this? Or how do you build a mystery tube? So they were very logistical and pretty specific. And our role initially, at least my perception was we needed to field those questions and we needed to communicate those to the PIs so that they could be aware of maybe corrections or clarifications to the curriculum, fill some holes. **There was discussion about pedagogy as well, but I think more so at the beginning than now we really paid attention to logistical things and details to help iron out. Now everyone who's in the project for the most part is pretty experienced with the curriculum. So that's less of an issue.** [Table 3, Quote 9] |
| 4:57 | Interviewer | So it sounds like, if I understand right tell me, that you initially, your role may have been to be a little bit more of a... You didn't use this word, but if an expert to help people who were newer to the curriculum to figure out sort of the practical challenges associated with implementing the curriculum and that now it's shifted and you see... So it sounds like you see yourself as a mentor, but initially it was mentor in this very like, "I've got some information that will help your way." And then now it's more mentor in a, "We're all kind of in this together to explore ideas of pedagogy way." Does that? |
| 5:34 | Craig | Yeah, yeah. |
| 5:35 | Interviewer | Okay. And so related to that, what would you say your goals are? What do you hope is accomplished in the meetings? |
| 5:45 | Craig | Yeah. So that's an interesting question. Because I'm, do you mean, what do you think or what do I think the PIs goals are? Or what do you think my personal goals or the PIs goals? |
| 5:57 | Interviewer | Your personal goals as a facilitator. |
| 6:00 | Craig | So my personal goals what I want to get out of this is just being involved in a project. I'm at a small regional institution. So I don't have a large research group just down the hallway or... |
| 6:16 | Interviewer | Yeah. And I guess, so let me clarify. This would be your goals in your role as a facilitator. So by the way, what do you hope is accomplished with the group that you are charged with facilitating? |
| 6:29 | Craig | Well, first of all, I want to make sure that their needs are being met or that they're able to that they're prepared to go on and do what they need to do in the class. So I want to make sure that everyone is... I guess that's still cycling back to the original purpose. I want to make sure that they're ready to go and that they don't have any immediate needs, but at the same time I really I'm looking to be a part of that communication in networking with others. So I guess I was kind of a... There's a bunch of different ways to answer this. I guess if I'm going to schedule a meeting and we're going to have a meeting and I've got to kind of write down the goals. First, I want to check in and make sure everyone's up and running that there aren't any significant issues. And then now I guess the goals are, let's talk about what we can do as a group and kinds of pedagogies we can talk and explore together, and maybe even generate some ideas for a presentation to give to AAPT. So it's... I don't know, the goals are more interacting and moving forward as like a little sub-community within my group. What kinds of tasks or challenges can we take on, aside from just talking the nuts and bolts. I don't know, kind of struggling with that question. |
| 8:10 | Interviewer | What would you hope that the participants would get out of their participation in a FOLC with you? |
| 8:18 | Craig | **Well, I would hope just like me that they would have an opportunity to share some things that they've come to realize, maybe an epiphany or something that they've developed in their own class, give them an opportunity to share some of the things that they've developed.** [Table 3, Quote 7] **And then to kind of unpack that a bit, bounce that around between the people who are in the group and give other people ideas. So from what members of the group can share, maybe that can generate some further discussion and further ideas as to how to improve the curriculum and just give people an opportunity to think about what it is that they're doing, not just from activity to activity, but in the grander scheme of things, in terms of pedagogy and improving their own teaching.** [Table 3, Quote 10] |
| 9:12 | Interviewer | Okay. So let me see, let me see if I can summarize here. You said several things in there. One was to check in with them on issues that they might be having. And then also for them to, I guess, talk about and get feedback on that. And then you also talked about giving them the opportunity to share things that they might be doing. So not just asking for help, but sharing things that they are doing and figuring out in their own classroom and then sort of on a higher level unpacking that and thinking about that in order to relate it into deeper pedagogical ideas. Does that? |
| 9:59 | Craig | Yeah, that sounds about right. There's also a level of decompressing. One strategy is to just talk about us, so what are some highs and lows that have happened this week and just give a chance for people to decompress. And that's tied into how I started this, which was, I think going in a different direction, but to serve as community and help each other wrestle with maybe not having others, that we can just go down the hall and talk to. This is... Here in these meetings, we've got people who are teaching the same exact curriculum and kind of a common mindset. |
| 10:39 | Interviewer | And so when you said, so one of the goals that you mentioned was for them to share their ideas and then as a group to do some unpacking and relate that to deeper pedagogical ideas. What would you say are characteristics of a good conversation around that? If you were going to tell me, view this conversation, and these are key markers that would indicate to you that you are doing that in a way that was productive, what would that be? Or who would it look like if it went well? Does that make sense? |
| 11:15 | Craig | Well, yeah. So one indicator would be that we've got multiple people who are chiming in, multiple people sharing their thoughts ideally without prompting. That's where we kind of fall into the facilitator roles. **If we see somebody who's not really talking, then we can pull them in and say, "So Susie, what's your take on this?" So one key indicator is that we've got everyone who's there engaging, and then sharing their experiences.** [Table 3, Quote 8] And I think another indicator is that an indicator of success or that things are progressing is that multiple solutions or multiple interpretations are brought forth. So it's not just, "Well, this happened in class, what do you think?" And then somebody just gives a flat answer, and then we move on to the next thing. So disagreement is okay and different ideas. I mean, different ideas or interpretations, that's a key marker for a successful meeting. |
| 12:23 | Interviewer | And what have you learned about how to nurture that, to make that kind of conversation more likely? It doesn't happen all the time, right? |
| 12:38 | Craig | Yeah, it doesn't. **One thing that I've learned, and I can call myself out and I know other facilitators or group leaders have done this too. We fall victim to this. We kind of have to put ourselves, our teacher mode in check, because we so often, we've got things that we want to share and, "Oh yeah, I've seen this before and this is what I did, and this is how I solve that problem." So we've really got a hold back on that as a facilitator and sometimes even if we're not a facilitator or a group leader, we need to give everyone space to talk and we can kind of, not that we can't say anything, but really need to make sure that we don't overpower the conversation with one idea.** [Table 3, Quote 5] Because that lends to the perception that there's really one solution to this. And they're often, I really don't think that that's the case. I think there's multiple ways to approach challenges. Getting back to the question, for me, **what I've learned is I've got to keep myself in check and make sure that I'm not dominating the conversation. Sometimes that means that even if I've got a great idea for the mystery tube, I actually don't get to share it. I'm more trying to get other people to talk about what it is that they've done.** [Table 3, Quote 4] |
| 14:04 | Interviewer | Anything else? So that's sort of passive, right? Holding back from projecting too much. Is there anything that you've learned that would be taking a more active role that helps nurture those kinds of conversations? |
| 14:19 | Craig | I think Next Gen PET [FOLC] has kind of transitioned to trying to demonstrate to the people in the community that they don't necessarily have to have a facilitator; you can engage in these conversations on your own. And then with the different spec channels and different projects, they tried projects one year, and I don't think that it was an overwhelming success. But I think one thing that it did do well was demonstrated that you can take on projects, even if you're doing your own thing and who knows where and regardless of the size of your department. There are things that you can do, projects you can take on and you can collaborate pretty easily. And it's just a matter of finding the right match for you, right collaborator. I don't know if that quite is getting what you're after? |
| 15:14 | Interviewer | Yeah. I think that's fine. Do you think over time, as you facilitated that what you're doing as a facilitator has changed in that way? |
| 15:30 | Craig | Yeah. B**ecause at the very beginning it was very much just the leaders were leading the meeting and at some point we had almost a bank of questions that we could draw from, if there was a low in the conversation and now it's much more organic conversation and we still have those prompts, but either through experience or just because of the whole group growing as a whole, I think that the conversations are more organic.** [Table 3, Quote 11] The one negative thing about those projects was that it did kind of force the hand in terms of the direction of the conversation. It's like, "Okay, we need to work on this project," but that's the nature of working on a project is it's got to be focused. So... silence that. So yeah, the role has changed, and I've kind of gotten myself derailed. I don't know. Does that answer what you'd asked? |
| 16:35 | Interviewer | I think so, yeah. And then the next question I have, I think you've already answered. It sounds like in the beginning that there was more of a focus on what you call sort of the nuts and bolts, and you might be a little bit more, interject a little bit more on helping them provide solutions and solve things, and that you shifted now to more of a focus on sort of deeper pedagogical discussions. And it sounds like you're taking a little bit more of a hands off so that they can interject their own ideas and not be dominated by yours. Does that sound right? |
| 17:17 | Craig | Yeah. And I think it's demonstrating that we're the leaders or **myself, I'm looking to learn from them just as much as maybe they're learning to look from others. But that's kind of how my style has evolved, not just in NGPET but in other areas as well as it's less as the top down and more as the "We're in this kind of as a group together and there's kind of breaking down the hierarchy of things.**" [Table 3, Quote 2] |
| 17:53 | Interviewer | And so... Let's see. So you talked a little bit about... I think you're going to answer probably my next question as well. So in terms of getting those discussions to happen, it sounds like the main things that you do are you talked about sort of withholding a little bit to try and give them the space to have their own conversations. I think that's probably this was the main thing that you talked about with how you would achieve this. Did I miss anything? |
| 18:38 | Craig | I don't think so. Again, we can use those prompts to kind of seed the conversation. But I don't know, there were occasions where I would use the highs and lows, so what are the highs or are there particular lows? And even if they weren't Next Gen PET related, so we could talk about something that happened in another class that was really kind of a great moment. And then we can say, "Well, okay, so what were the conditions that set that up?" "And is that something that happens regularly in Next Gen PET or other courses, or how can we help that, keep that happening in other courses in other contexts?" So I'll use those highs and lows as a way to seed the conversation. And then once people start talking about something, then I'll just kind of let it go until they get to say, we'll kind of go around the circle and make sure everyone has a chance to speak. |
| 19:42 | Interviewer | And how satisfied are you with the quality of the conversations that are happening? |
| 19:47 | Craig | So it varies. I mean, sometimes we'll have a better week than other weeks. In general, I'd say I'm pretty satisfied. I think my perception is that people are getting what they need out of the meetings because they keep coming back and they're contributing. I mean, if you're not getting what you need you stop going if it's not worth it. The fact that people keep coming back and having these conversations and getting to know one another, and then the conversation is a little bit more relaxed and natural free-flowing. |
| 20:24 | Interviewer | Okay. Do you feel like you've gotten better over time? |
| 20:31 | Craig | I would say so. Yeah. In terms of contributing and helping lead the meetings, from when I first started, I was very much looking at my old board and kind of making sure I'm going [with] those questions and getting them all in and then taking notes as we're going along. It was very mechanical and now it's less mechanical and of course there's still structure, but it's more a conversation. |
| 21:02 | Interviewer | And do you think that that is a reflection of you and how you've kind of learned and grown as a facilitator or, I mean that the audience has sort of changed because they were novices in the beginning and now you've been together much longer and they all, as you said, are pretty expert like themselves now. |
| 21:24 | Craig | Yeah. So an interesting question, and a way to answer that would be, if I were to start doing this again, with a completely new audience, I think my initial tactics would be quite different. I think well, they might need some of those nuts and bolts. I think the conversation, I mean, so to answer your question briefly. Yeah. I think I would change quite a bit. I think prior to start where I started before knowing what I know now, I would be much more comfortable entering in those dialogues and helping people kind of contribute in those conversations. |
| 22:05 | Interviewer | Do that. This is really interesting. What would you... Can you help me understand what you would do differently? |
| 22:11 | Craig | Well, I think it's just being able to have a conversation and instead of being like I ended up before mechanical going down question by question, being a little bit more receptive to... I don't know when somebody says something has happened in class instead of saying, "Oh, well, that's interesting. What do you think about that?" Now I can draw on other experiences and say, "You know, I remember somebody else having a similar experience and this is what they tried and it didn't work or it did work." So I've got more to draw on. And instead of just a quick pass to somebody else, I might interject something and then kind of seed the conversation further. With novices you might need to do that because they don't have a whole lot of experience to draw from. When working with novices and I wasn't where I am now. I didn't necessarily have that capacity, that experience. |
| 23:09 | Interviewer | So it sounds like what you feel like is that what would be different would be that you just have more sort of content knowledge, I guess, as such to be able to seed into conversations with them. |
| 23:23 | Craig | Yeah. **Content knowledge and... So there's something else though. It's not just the content knowledge, it's just the experience of working with this community and the other faculty who are teaching the same curriculum. So often we're in our silos and we're doing our own thing. And we're used to doing things on our own. I think it's being able to pull out of people, what it is they really think about, what their real thoughts are on a particular issue or item rather than just a superficial treatment and being able to get a little deeper.** [Table 3, Quote 12] |
| 24:09 | Interviewer | Okay. What would you say has been most challenging about being a facilitator? |
| 24:23 | Craig | So making sure that I am along with the other facilitator, making the time spent worth the other people's time. That's probably my greatest worry, is I just don't want to fill a meeting just to fill a meeting. Sometimes there's just not a whole lot that's going on and the group isn't very talkative, so to speak. So our greatest challenge is just to make sure that we're having a dialogue that's worth having, rather than just stuff that I don't know, I have a big fear about just wasting people's time. So making sure that we're moving forward with some kind of productive discussion. I mean, that's... You said the greatest challenge and I guess that is a challenge of mine, but I wouldn't say that, that's dominated this experience, because really it's getting the conversations going hasn't been horribly difficult. But when there is a lull in the conversation and people are kind of that's just a challenge of mine is make sure that we're not wasting people's time. |
| 25:39 | Interviewer | And have you... Are there any things that you've learned, like if you could think of a time when there was a lull in the conversation and you thought, "Oh, I don't know if this is worthwhile. Do you have any strategies that you've learned for those moments? |
| 25:57 | Craig | Yeah. So if there's a lull and it just kind of it's tapering off, then we'll say, "Well, we're on the Slack channel, you've got our email and it seems like we're kind of fading off in terms of productive conversation. So we just might end the meeting early," and instead of forcing it to fill the time, just say, "I think we've had a really good meeting up to this point and if there are pressing issues or if we've kind of exhausted everything for the moment, then jot stuff down and the next week, and we'll get back at it the next time we meet." |
| 26:28 | Interviewer | I'm guessing you have hadn't that happen in the first 10 minutes or something, right? |
| 26:33 | Craig | Oh, no. It's usually after 35 minutes or so, 20 minutes maybe, but usually the first 10 to 20 minutes, are pretty engaged. |
| 26:47 | Interviewer | Okay. |
| 26:48 | Craig | I would say that ending a meeting early it's probably only happened two or three times. So I don't know why I worry about it so much. |
| 26:57 | Interviewer | Yeah, I was going to say, it sounds like it's more of a worry that you have than something that you're being an actuality. That's just sort of like wanting to make sure you're doing a good job. |
| 27:07 | Craig | So if I reconsider that, what are the greatest challenges? I mean, aside from the logistical thing of making sure that we get something scheduled but that's not really a sick, that's just a... I don't know. I mean, I've not really seen this whole project as being a challenge. I've seen it as being an opportunity. And there's been some challenges where we've got a participant and I suppose we're guilty of this too. **Sometimes the leaders can get into a mode of dominating conversation. And so a challenge can be to, try to hint or redirect so that it opens the space for everyone. Because you've got people who are in that teacher mode and they want to share what they've done and they've got their solutions. So that's a challenge from time to time**. [Table 3, Quote 3] But yeah, so I think you're right. I think you identified, it's more of a worry with keeping things productive. |
| 28:23 | Interviewer | Okay. So let's shift a little bit then. What's it been like having a co-facilitator? |
| 28:29 | Craig | Well, I mean, for the most part it's been great, because there've been some weeks where something's popped up and I've not been able to be there. And so the co-facilitator takes over. And then being able to, when we can meet a little bit before the meeting or a little bit after the meeting, just to kind of maybe come up with a theme or decompress a little bit about what happened and then talk about how maybe we want to approach that in either the Slack channel or next time that we meet. It's nice to be able to bounce ideas off of somebody else, who's kind of filling the same role as you, just kind of like [co-facilitator] was a co-facilitator just to, so "[co-facilitator] , what was your takeaway there because I'm curious?" And then I'll talk about my takeaway and we can go from there and say, "Well, is it, we just leave it as it is or is that something worth putting on the agenda for next time that we meet?" |
| 29:28 | Interviewer | So it sounds like with your co-facilitator, that they were helpful. I think you identified two things. One was, just kind of helping take the burden off a little bit of taking the time involved, but then also just having that other person to bounce ideas off of, and I'm imagining both in terms of planning what you're going to do and in real time, as you're trying to navigate the discussions. |
| 30:00 | Craig | Right. |
| 30:00 | Interviewer | Okay. And what about the group members? Did they play a role in helping you to get good discussions going? |
| 30:16 | Craig | So group members, you mean people who are in the... |
| 30:16 | Interviewer | Let me try and ask the question a little bit differently. So what we're really interested in is some I'm interested right now in the kinds of things that have influenced your facilitation and influenced your ability as you were sort of learning and growing as a facilitator and what you do. And so, there's your co-facilitator, but then there's also the group. And so what I'm curious about is the role that the group members might've played in terms of helping you be a facilitator grow as a facilitator, meet the goals that you have of these good deep unpacking kind of discussions. Does that make sense? |
| 30:53 | Craig | Yeah, I think so. One of my faults is that, I don't check Slack as regularly as others, and I know that there's a lot of discussion that goes on with Slack. And so I've pulled up occasionally and I'll try to get myself up to date. I've just not met, that's just not part of my daily routine. I do know that the group is there to support other people whenever they need them. And just knowing that that support is there, has been a great help. Because I know that if I ever have an issue with something that's technical or just a detail, like accessing a video, I can get the help right away. Or if I've got an idea for an engineering design project, there are a lot of people that are interested and willing to help out. And so just knowing that the support is there is great. So it was probably something, I don't know, maybe the other facilitators feel the same way. It's probably under utilized and the support that the group has to offer. But I know that a lot of people have leaned on other group members. |
| 32:11 | Interviewer | Okay. And it sounds like in your groups, that they the participants take a lot of initiative in terms of directing the conversations and seeding them that you don't feel like that's all up to you, that they also take on a lot of that as well. |
| 32:28 | Craig | Right. |
| 32:31 | Interviewer | And I'm guessing more over time, have you seen that increase over time from the participants they're sort of? |
| 32:38 | Craig | Yeah, yeah. And then the PIs also, they have their ideas for... I mean, I think they're doing a great job because they each year of this project they've said, "Okay, so this year, what we're going to do is we're going to try doing this." So as a facilitator kind of keep this as in the back of your mind. We want to focus on really getting at pedagogies and how this can push forward and maybe spill over into other courses and that sort of thing. So, yes, to answer your question, the participants in the group are contributing more. At the same time, the PIs are I think, aware of that, but at the same time they're also providing backup and some direction just to I don't know, maybe it's overarching objectives for the groups to consider at least explore. |
| 33:37 | Interviewer | And so along those lines, they gave you some facilitation guidelines in August of 2019, and then some planning, reflections, suggestions in January 2020. Do you remember those? |
| 33:53 | Craig | I remember getting documents from them. And I'm looking at my work because at one point I had it tacked up to my bulletin board, which is right behind my monitor. So I remember going through those, especially the questions. And then I don't know that we really wrote a whole lot of reflections. I mean, we would talk with the facilitator co-facilitator and I would talk about what it is that happened. And then we would take notes in Slack or in a shared Word document. So trying to push that back out to the people in the group during the meetings, we would kind of reflect on things, but there wasn't anything that was necessarily officially produced. |
| 34:40 | Interviewer | So did you find it helpful, the stuff that they gave you for the meetings? |
| 34:47 | Craig | I did. Because at the very beginning again, things were pretty mechanical, but then as the project advanced and grew older, people were no longer novices. So I think it provided guidance as to where we could go aside from just talking about, "Are you short on supplies, has a textbook come in and stuff like that." So it's been helpful. |
| 35:15 | Interviewer | So it sounds like it helped you kind of move beyond the sort of more superficial conversation into some of these deeper things that are more interesting. What else has helped you develop as a facilitator? |
| 35:37 | Craig | I think just being a part of the community and getting to know people. I know from year to year, we would change groups and actually we're in the current cycle now is in making sure we establish new groups. And so getting to know different people and just becoming more familiar with them, it's not like a stranger coming in and leading our group now, now these are people that I've worked with on different projects. I've seen them at the FOLC virtual conference. So we're just kind of getting to know one another and even at a personal level. So that's been helpful just being a part of something and knowing the group a little bit better. |
| 36:30 | Interviewer | Have you had much interaction with other facilitators other than your sort of official co-facilitator? Have you discussed facilitation with other facilitators besides your official co-facilitator? |
| 36:44 | Craig | A little bit, there hasn't been, at least with me there hasn't been a whole lot of chatter [?] in terms of, "Here are my experiences as a facilitator. What are your experiences as a facilitator?" There hasn't been too much of that. I do know that we did have group meetings [facilitator and project staff meetings] and I was not able to attend all of those. So there were opportunities to have those kinds of discussions. But outside of those structured conversations I haven't had those specific conversations with very many facilitators. |
| 37:23 | Interviewer | It sounds like you also had a lot of challenges where you felt like you needed to get support. It seems like you felt like you had the support that you needed. |
| 37:32 | Craig | Yeah. There was one time when we had a participant who was really struggling with an issue and it dominated one of our discussions and spilled over into the following time that we met. And so the co-facilitator and I, we chatted, we met, I think by Zoom. And then through email correspondence, we really talked about this one participant. So we pulled that conversation off to the side and visited with that person through email and trying to help them come up with potential solutions. I think a lot of what they needed was an opportunity to vent and the meetings can serve some of that, but we felt like it was starting to spill over and be too much of a thing like... So yeah, we transitioned to email communication with that particular participant, but that was kind of an isolated case. And that's where the facilitator and I really kind of bonded a little bit like, "Oh, what are we going to do here? We kind of feel for this person, but at the same time, we don't want it to completely overtake what it is that we're trying to do with the group." |
| 38:45 | Interviewer | And are there things that you think the project team could do to better support the facilitators? |
| 38:54 | Craig | Well, I mean [crosstalk 00:38:56] |
| 38:56 | Interviewer | And if the project where we're starting with new people. So also kind of going back to the beginning. |
| 39:03 | Craig | So time is pretty precious. I can say this, but at the same time, I know that it might not be able to be worked out. And I know that they've offered these opportunities but to have meetings among facilitators, like Zoom meetings among facilitators might be helpful, but we're already doing a meeting. Well, depending on the group, a meeting every other week. And there were times when I was doing two meetings per week to help accommodate people's schedules, which was fine. I'd agreed to do that. So to have an additional meeting among facilitators, that it could be kind of a hardship or it might be difficult for people to actually attend, but that could be helpful. And again, it was kind of a constraint of my own schedule, not being able to attend as many of those as were scheduled. But, I don't know. I see the improvements as much as my own responsibility, maybe more so my own responsibility than the PIs, because there are things I could have done if I were able to attend those programs or those group meetings, whenever they were offered and if I had a routine of checking into Slack more routinely and then that could have helped that. |
| 40:33 | Interviewer | And they're going to be trying to produce a guide for new facilitators, based on your experience what advice would you have to go in there? What do you think might be included in there besides the obvious stuff? What might be good to kind of put in there as advice to new facilitators? |
| 40:59 | Craig | Know that you don't have to answer questions as they're asked. We can always... of course, I guess as faculty we know this, we can always say, "Well, let's look into that. I'll look into that for next time." But you've got to make sure that you follow up with it and some communication can happen through back channels or through email communication. It doesn't have to wait the whole two weeks before your next meeting. So different people communicate. So this is kind of like a separate issue or separate item, but different people communicate different ways. There've been some times where the group was all in with Slack. And then there were times where nobody used Slack. And so it was sending email out. So the facilitators need to be flexible and recognize that the people who are in their group they may not use Slack or they may not respond to email. We've got to figure that out. And so, either go one way or use multiple. And I don't know that that might not be what you're getting at. Because you're after some really kind of novel things to put in that guide. |
| 42:17 | Interviewer | Not necessarily I'm interested in what comes to mind for you. What do you wish that you had known, right. If you could go back and give advice even to yourself in the beginning, what do you wish, what do you think would have been helpful to have known that you didn't? |
| 42:36 | Craig | Well, I can start off with something that I did know or that I kind of, what motivated me to become involved was I saw this as an opportunity, an opportunity to get involved professionally in a way that I wouldn't be able to get involved with otherwise. And so recognizing it as an opportunity and then treating it as though it's an opportunity. So it's really a privilege to be involved in this project. And so moving forward with that kind of in mind or framing it that way, you're more apt to take advantage of or become full. Let's see to become more engaged authentically with the project, rather than it's something I've got to do, check this off week after week or every two weeks. So other things that I wish I had known getting back to your question, know from the get go that people are there to support you and that they're willing to work on projects with you and collaborate. So this is tied to that opportunity. I saw it as an opportunity for me to kind of get involved with something, but I didn't necessarily fully realize the extent of the opportunities. I think the PIs are interested in helping people accomplish things professionally. I think you've got a bunch of other faculty and they're super busy, but you know what? If you were to collaborate with them, then maybe you could churn out a paper or do copresent at a meeting. It's an opportunity but at the same time, a lot of people are in the same boat and they're just as eager to team up with people and work on projects too. |
| 44:24 | Interviewer | Okay. And then anything that you would differently, if you could do it all over again? |
| 44:39 | Craig | I don't know. I've really enjoyed the project. I think I tried to engage as much as I could, we had the where we actually had the projects where we were supposed to take something on and I got involved with a project with, let's see, and I don't know that anything came of that necessarily. There was another project that we got involved with [member] and [member]. And then there were some others that were involved that actually resulted in a paper. I mean, I've gotten a lot out of this, so I don't know if there's a whole lot I would do differently. I might work harder to set up a routine to check Slack and kind of be more engaged, but that was a time constraint. There's simply not enough time for me to check Slack, like checking social media. I don't know. So I'm bouncing all around you kind of fishing for something that I'd improve or that I would do differently. |
| 45:45 | Interviewer | There could be nothing. There doesn't have to... |
| 45:48 | Craig | Yeah, I don't know. The main thing would be just keep a better pulse on making sure that I'm communicating with... Because there was part of the project kind of fell through and I felt bad about that. And I think it was just because I got so swamped with things that I wasn't even able to communicate promptly enough or timely enough to contribute as much as I would've liked. But that's not something that is necessarily in my control. The time was just really pressed at my end. |
| 46:34 | Interviewer | My final question, is there anything that you have learned as a facilitator specifically that you find yourself applying in other domains in your life? So outside of this project and outside of teaching Next Gen PET? |
| 46:58 | Craig | Well for a while now I've always had this philosophy that there's more than one solution, and there's more than one right way to tackle things, serving as a facilitator and listening to people, going through these conversations, it's just kind of reaffirmed that. And so I can... assertive is the wrong word, but I can be more, I can apply that more easily now than I could have before. Department chair also at my institution and so going to a meeting and people, they bring up an item and it's up for discussion. 10 years ago me would have really honed in on a single solution and now it's like, "Okay, well here's my idea for what might be a solution, but let's hear everyone's idea. And then, I'm not opposed to just completely getting rid of my original idea and going with either kind of a conglomerate of what other people are proposing or somebody might just have like the greatest clear cut solution. I don't know. I don't feel always, so that's quite what you're getting after. But walking into a meeting, I feel as though I can engage in the meeting as a participant, but then if I need to it's easier for me to switch into more of a leadership role and say, "Okay, no, let's bring this back. Let's cycle back to the original item at hand." |
| 48:41 | Interviewer | Sounds like if I sort of paraphrase that you've learned maybe when you're, even like, as your conversations is your chair to do a little bit better at recognizing and welcoming more of a diversity of ideas and solutions than maybe you had before. |
| 48:59 | Craig | Yeah, yeah. And yeah. That's accurate. I would also say that it's less about when the meeting starts, we start the meeting and it's all just, line item by line item it's you're checking in and kind of getting that personal side. I mean, sometimes in a departmental meeting or a chair meeting or something like that, there's not very much of that that goes on, but if I'm in a committee something that I've learned is that, if you can make a little bit of a personal connection, it doesn't have to be all touchy feely, but if you can make a little bit of a personal connection with committee members the productivity can really improve quite a bit. Because you're... I don't know, it's a more cohesive group. And so I guess that's one thing that I can take away from this is learning how to try to make our group more cohesive rather than just going through items on an agenda. |
| 50:03 | Interviewer | Sounds like a nice thing to have learned. It sounds like a nice thing to have learned. |
| 50:08 | Craig | Yeah. |
| 50:10 | Interviewer | Those are the end of my formal questions. Is there anything else that comes up for you that along these lines that I might be interested in that I haven't thought to ask? |
| 50:25 | Craig | I don't know. There were some outcomes of being involved in this project that I'm really glad I got involved. Because I remember visiting with it was Ed and Fred. It was at an AAPT meeting and I either caught wind of this FOLC group or I just happened to talk to them. And I said, "You know, if you are getting involved in anything with PET, kind of drop me a line because I'm interested." And that was just a conversation. I think it was with Fred that I had that. And he just kind of Fred and he just kind of smiled and nodded and said, "Oh yeah, okay, well maybe," and I don't know how long it was later, but I got an email and I'm glad that they've reached out to me. And that's how I got involved with FOLC, in the Next Gen PET program. And I don't know since that time, I mean, I was able to publish a paper in the PET proceedings and then I felt kind of empowered to create some videos for the virtual conference, which was great timing by the way, because that ended up being good training for the AAPT conference. And, it kind of, it was something that I had been wanting to get into anyway, which is getting a little bit more proficient with making videos. And it just kind of forced my hand, so to speak to get involved with that, but it was great timing and I enjoyed making those videos and then it helped me for AAPT. So I've really been fortunate with being involved in this project. I think it's helped me out different aspects quite a bit. |
| 52:12 | Interviewer | Awesome. Well, thanks for taking your time today to talk about it. |
| 52:15 | Craig | Yeah, yeah. Well, thanks. And I'm sorry that I was kind of slow to log on. I appreciate you- |
| 52:20 | Interviewer | I was sitting here doing stuff and that's what I'm going to keep doing. So it doesn't matter in the least. Yeah. Thank you. I appreciate it. And have a good rest of your day and hopefully things will go well with the... How do you do interactive or you just don't you just not have the students interact with each other when they're six feet away? |
| 52:41 | Craig | Well, so I really struggle with that, my physics class is pretty small. And so it's a real small group and it's a small room and so we have discussions and that works out okay. But yeah, I've got a larger class like earth and space science and they're 20 students and they're all spread out in this amphitheater. And yeah, so I use clickers or have in the past and the whole purpose of the clickers was I pose a question and say, "Okay. Here's where we are, now turn to your neighbor and talk." Well, I don't do that now because I don't want them turning to their neighbor and talking. And so it creates some awkward moments because we'll get to a slide and I'm like start to say, "Okay, talk to your neighbor." And so I struggle with that. So now I call on people kind of at random. And of course they've got the phone or friend. So if they get stuck, they can point to somebody and they can help them out. But cannot really do the peer instruction like I'm used to. |
| 53:49 | Interviewer | It almost seems like it would be better to just be virtual because then you could put them into small groups. |
| 53:56 | Craig | Yeah. So that's interesting. It's kind of like this diminishing returns, where you're forcing the face-to-face, but they're actually diminishing returns because of the physical distancing. But that's where we are. We at my institution, we've got the option to do hybrid. So it's required that we meet with students face-to-face once per week. But beyond that we can do things through Zoom or remote learning. So I don't know, maybe that's something to consider. Because I have that option, but I don't know for like a lab, it would be difficult because you've got to work with manipulatives, but yeah. I'm glad you said that it was... |
