# Supplementary material for: Understanding how facilitators adapt to needs of STEM faculty in online learning communities: a case study
Source: Int J STEM Educ. 2022 Sep 5;9(1):56. doi: 10.1186/s40594-022-00371-x (PMC9443628; doi:10.1186/s40594-022-00371-x)
Supplement: Supplementary file 6 — Additional file 6. Supporting quotes for three change themes. [file 40594_2022_371_MOESM6_ESM.docx]

**Supplemental Material: Supplementary Table 1**

[Supporting quotes for the three change themes identified in the case facilitator’s interview]

**Table 1**

*Three change themes and supporting quotes from the interview of the case study facilitator*

| Change Theme | Supporting Quotes from 2020 Interview |
| --- | --- |
| **Role Change**  He originally believed his role was to draw on his expertise by sharing his own experience with the group. Later, he believed that his role should be to share his experience less frequently and draw on members’ ideas and experiences more often. | 1. “...reflecting back and kind of retrospectively now and seeing myself as serving as a mentor, but also others are mentoring me as well. It's [...] much less explicitly directed by one person who's leading everything, leading the charge. And now it's more like we're trying to get a conversation and a dialogue going, where it's not just the leader that has things that he or she, or they need to share. It's everyone collectively.” 2. “I'm looking to learn from [the members] just as much as maybe they're learning to learn from others. But that's kind of how my style has evolved, not just in NGPET but in other areas as well, as it's less top-down and more as the ‘We're in this kind of as a group together and there's kind of breaking down the hierarchy of things.’” 3. “Sometimes the leaders can get into a mode of dominating conversation. And so, a challenge can be to try to hint or redirect so that it opens the space for everyone. Because you've got people who are in that ‘teacher mode’ and they want to share what they've done, and they've got their solutions. So that's a challenge from time to time.” 4. “What I've learned is I've got to keep myself in check and make sure that I'm not dominating the conversation. Sometimes that means that even if I've got a great idea for the mystery tube, I actually don't get to share it.” |
| **Response Change**  He changed his practice by jumping-in to address issues raised less often, and withholding his own response more frequently, to encourage member sharing. | 1. “We kind of have to put ourselves, our teacher mode in check, because we so often, we've got things that we want to share and, ‘Oh yeah, I've seen this before and this is what I did, and this is how I solve that problem’… So, we've really gotta hold back on that as a facilitator [...], we need to give everyone space to talk [...] I'm more trying to get other people to talk about what it is that they've done.” 2. “I see my primary role as [...] just helping organize discussions, lead the discussions to some extent… When we first started, [...] we very much led and then, as we continued with the program or the project, it was, "How do we get the others, the other people who are in the meeting to help lead the discussion?" 3. “I would hope, just like me, that [members] would have an opportunity to share some things that they've come to realize, maybe an epiphany or something that they've developed in their own class, give them an opportunity to share some of the things that they've developed. And then to kind of unpack that a bit, bounce that around between the people who are in the group and give other people ideas.” 4. “If we see somebody who's not really talking, then we can pull them in and say, "So Susie, what's your take on this?" So, one key indicator [of a good meeting] is that we've got everyone who's there engaging, and then sharing their experiences.” |
| **Meeting Content Change**  He observed that initially the FOLC group discussions were more logistical in nature, whereas later the group members could engage in more pedagogical discussion. Consequently, he changed his practice to promote opportunities for pedagogical conversation. | 1. “Initially I think it was very nuts and bolts and logistical. [...] There was discussion about pedagogy as well, but I think more so at the beginning than now we really paid attention to logistical things and details to help iron out. Now everyone who's in the project for the most part is pretty experienced with the curriculum. So that's less of an issue.” 2. “[I hope that] from what members of the group share, maybe that can generate some further discussion and further ideas as to how to improve the curriculum and just give people an opportunity to think about what it is that they're doing, not just from activity to activity, but in the grander scheme of things, in terms of pedagogy and improving their own teaching.” 3. “...at the very beginning [...] we had almost a bank of questions that we could draw from if there was a lull in the conversation, and now it's much more organic conversation. And we still have those prompts, but either through experience or just because of the whole group growing as a whole, I think that the conversations are more organic.” 4. “Content knowledge helped [the discussions, but] … it's not just the content knowledge, it's just the experience of working with this community and the other faculty who are teaching the same curriculum. [...] I think it's being able to pull out of people what it is they really think about [...] a particular issue, rather than just a superficial treatment, and being able to get a little deeper.” |

*Note.* Quotes are identified in bold in the Case Facilitator Interview Transcript (Supplemental Material)
